# The Mystery of Dibenzoterrylene: A Clar‐interlocked Biphenalenyl Biradical

**DOI:** 10.1002/chem.202502952

**Published:** 2025-11-10

**Authors:** Elena A. Kalinina, Mikhail A. Kalinin, Dmitry I. Sharapa, Harald Maid, Alexandra Freidzon, Haleh Hashemi Haeri, Dariush Hinderberger, Robert Dinnebier, Alexander S. Oshchepkov, Konstantin Yu Amsharov

**Affiliations:** ^1^ Organic Chemistry Department Martin‐Luther‐University Halle‐Wittenberg Halle 06120 Germany; ^2^ Department of Physics Max Planck Institute for the Science of Light Erlangen 91058 Germany; ^3^ Institute of Catalysis Research and Technology Karlsruhe Institute of Technology (KIT) Eggenstein‐Leopoldshafen 76344 Germany; ^4^ Institute of Organic Chemistry II University Erlangen‐Nuremberg Erlangen 91058 Germany; ^5^ Department of Molecular Chemistry and Materials Science Weizmann Institute of Science Rehovoth 7610001 Israel; ^6^ Institute of Chemistry Physical Chemistry–Complex Self‐Organizing Systems Martin Luther University Halle‐Wittenberg Halle 06120 Germany; ^7^ Max Planck Institute for Solid State Research Stuttgart 70569 Germany

**Keywords:** biphenalenyl, biradical character, DBT, PAHs, single‐molecule spectroscopy

## Abstract

The first time in‐depth characterization of dibenzoterrylene (DBT) was performed by a combination of various experimental and computational techniques. First structure confirmation and atom assignment were achieved by means of 2D‐NMR and powder X‐ray. DFT methods shed light on the conformational space of DBT and, more importantly, on the complex electronic structure of the molecule. DBT appears to be the first studied molecule, two low‐lying conformers of which have drastically different biradical character. EPR and NMR studies show the effect of solvent on the population of the biradical state. This demonstrates the perspectives of the usage of DBT and its derivatives in spintronics and other quantum technologies.

## Graphical abstract

We discovered a unique conformational switch that transforms a molecule from a closed‐shell to a pronounced biradical state. This geometry‐induced control of electronic ground states offers a design principle for stable, low‐gap open‐shell systems with potential applications in spintronics and responsive organic materials.

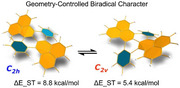

## 1 Introduction

Organic molecules have recently garnered significant attention for use in quantum devices due to their exceptional properties, versatility, and virtually unlimited tuning possibilities.^[^ ^1^ ^]^ Among them, polycyclic aromatic hydrocarbons (PAHs) stand out as some of the earliest studied and classical solid‐state quantum emitters.^[^ ^2^ ^]^ Due to their outstanding properties, PAHs serve as benchmarks in this field.^[^ ^3^ ^]^ In particular, PAHs encapsulated in organic crystals can generate high‐purity photons suitable for applications requiring single‐photon emission.^[^ ^4^ ^]^ These molecules emit photons with a narrow bandwidth, limited only by the lifetime of their excited state, making them excellent sources of indistinguishable photons.^[^ ^5^ ^]^

One of the most advanced PAH‐based single‐photon emitters in quantum technologies is 7,8:15,16‐dibenzoterrylene (DBT).^[^ ^6^, ^7^, ^8^ ^]^ When embedded in a crystal matrix, DBT exhibits high quantum efficiency and stable emission wavelengths.^[^ ^9^, ^10^, ^11^, ^12^ ^]^ Notably, Hong‐Ou Mandel interference experiments have demonstrated that DBT molecules deliver nearly ideal indistinguishable single photons at cryogenic temperatures.^[^ ^13^ ^]^ Furthermore, the emission properties of DBT can be enhanced through coherent molecule‐resonator coupling, allowing it to conform to a two‐level quantum system.^[^ ^14^ ^]^ Additionally, when integrated into 2D materials, its emission can be electrically controlled.^[^ ^15^, ^16^ ^]^ The substantial number of publications in this area attests to the ongoing and growing interest in DBT and its potential applications.

Although DBT was first synthesized by E. Clar in 1955, no other documented attempts at its synthesis and comprehensive characterization have been reported to date.^[^ ^17^ ^]^ A key challenge lies in DBT's limited solubility and stability in solution, which imposes serious constraints on its characterization. Prior to the present study, only a few spectral properties (e.g., UV and fluorescence spectra) had been reported.^[^ ^18^, ^19^ ^]^

In this study, we present for the first time a comprehensive characterization of the DBT molecule. Based on 2D NMR spectroscopy and powder X‐ray diffraction (PXRD), we successfully elucidated the connectivity and solid‐state configuration of DBT. A detailed DFT analysis revealed that the nonplanar geometry of the molecule plays a key role in the emergence of biradical character. Our findings suggest that DBT can be viewed as a Clar‐sextet‐interlocked biphenalenyl, in which the radical centers are localized at the molecular core, rather than the periphery of the π‐system. This unusual electronic structure results in a combination of significant biradical character and unexpected stability, even in the absence of bulky steric protection. Furthermore, we demonstrated that interconversion between DBT stereoisomers has a profound impact on its electronic structure. In particular, the *C*_2v_ stereoisomer exhibits a nearly tenfold increase in biradical character, accompanied by a thermally accessible singlet–triplet gap. Notably, by modulating the molecular geometry, the singlet–triplet energy gap can be tuned, enabling access to near‐degenerate singlet–triplet states.

## 2 Results and Discussion

We synthesized DBT following the procedure reported by E. Clar,^[^ ^17^ ^]^ with modifications to the purification steps as detailed in the Supporting Information (SI). The compound was found to be stable in the solid state but exhibited degradation in solution, with the rate depending on the solvent and exposure to light (see SI for details). This behavior is unusual for closed‐shell polycyclic aromatic hydrocarbon (PAH) systems and immediately drew our attention. Consequently, we undertook a more detailed investigation of this molecule.

**Figure 1:**
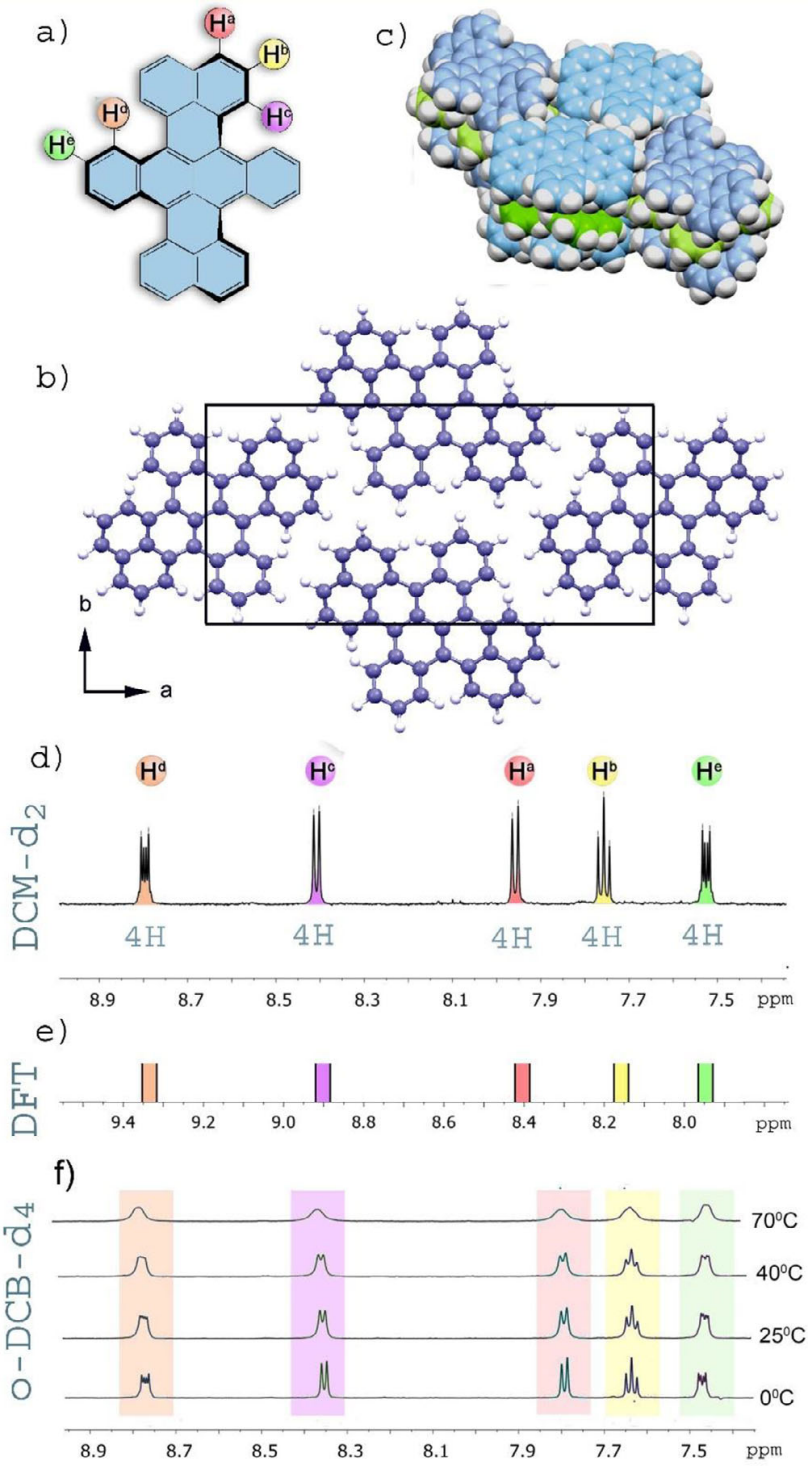
a) Molecular structure of DBT and color‐coded assignment of nonequivalent hydrogen atoms, b) projection of the DBT crystal structure onto the (001) plane, derived from PXRD data, c) Van der Waals model of a DBT crystal fragment, highlighting the dense slipped‐stack molecular packing, d) ^1^H NMR of DBT (DCM‐d_2_, ‐30**°**C), e) ^1^H NMR of DBT simulated in continuous DCM with PBE0/def2‐TZVP, f) a series of temperature‐dependent ^1^H NMR spectra of DBT in deuterobenzene and *o*‐DCB‐d_4_.

All attempts to grow single crystals from solution were unsuccessful due to the rapid decomposition of the compound. To overcome this limitation, we employed gas‐phase crystallization via slow sublimation under reduced pressure (10^−4^ mbar). The resulting crystals formed as thin needles, which posed challenges for single‐crystal X‐ray analysis. Nevertheless, we were able to determine the molecular structure, configuration, and solid‐state packing using PXRD. The molecules are arranged in a 2D layered structure within the a–b plane (Figure 1b). Despite their helical geometry, they adopt a dense slipped‐stack packing motif (Figure 1c), which facilitates effective π–π interactions—a packing arrangement commonly observed in the solid‐state structures of planar aromatic systems. Notably, the observed interlayer distance of 3.69 Å is significantly larger than that typically reported for planar PAHs, which usually ranges from 3.3 to 3.5 Å.

Although DBT has been known for a long time and has been the subject of numerous investigations, no NMR spectra have been reported in the literature to date. This absence is primarily due to the compound's low solubility and its rapid decomposition in solution under light exposure. Initial attempts to record NMR spectra in freshly prepared CDCl_3_ solutions yielded no detectable signals, likely due to immediate degradation. In contrast, spectra were obtained in freshly prepared solutions of deuterated benzene (C_6_D_6_), deuterated dichloromethane (CD_2_Cl_2_, DCM‐d_2_), and deuterated ortho‐dichlorobenzene (o‐DCB‐d_4_), as shown in Figure 1. The overall pattern of the NMR spectra in all solvents is quite similar and is well reproduced by DFT‐simulated spectra (Figure 1e), enabling a reliable assignment of the observed peaks. These assignments are further supported by 2D NMR experiments (Figure S11), showing excellent consistency between experimental and theoretical data.

Even more striking is the temperature dependence observed in different solvents. In deuterobenzene (C_6_D_6_), increasing the temperature had almost no effect on the spectral resolution. In contrast, in *o*‐DCB‐d_4_, the spectra became increasingly broadened with rising temperature.

**Figure 2:**
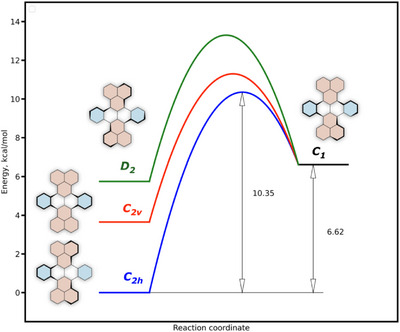
Relative energies and calculated activation barriers for interconversion between DBT conformers.

Generally, the broadening of NMR signals can be attributed to various dynamic processes, such as conformational transitions or molecular aggregation. A conformational search (B3LYP functional,^[^ ^20^, ^21^, ^22^ ^]^ def2‐TZVP basis set^[^ ^23^ ^]^ and Grimme's D3 dispersion correction with Beck‐Jonson's damping^[^ ^24^ ^]^) revealed that DBT can exist in four distinct conformers—designated by their symmetries as *C*_2h_ *, C*_2v_ *, D*_2_, and *C*_1_ – which are expected to be in thermal equilibrium at room temperature (Figure 2). Interestingly, our findings differ from those reported in,^[^ ^25^ ^]^ where the second‐lowest energy conformer—which we demonstrate to be crucial in later analysis—was not identified. Instead, that study reported an alternative *C*_2h_ conformer that is significantly higher in energy (see SI for a detailed comparison).

Figure 2 presents the relative stabilities of the identified DBT conformers, along with the activation barriers for their interconversion. The computed barriers indicate that rapid conformational equilibration occurs under ambient conditions. Notably, the energy difference of 3.7 kcal/mol between the two lowest‐energy conformers suggests—according to the Arrhenius equation—that at room temperature, over 99.7% of the molecular population adopts the *C*_2h_ geometry (in the absence of solvent effects). This conformer also corresponds to the structure identified in the PXRD analysis.

DFT simulations employing various implicit solvent models did not reveal any significant stabilization of one conformer over another (Table S3, S4), although it should be noted that these models do not account for specific solute–solvent interactions. Furthermore, the solvent‐ and temperature‐dependent behavior observed in the NMR spectra cannot be satisfactorily explained by conformational equilibria alone. The hypothesis that aggregation causes the signal broadening is also inconsistent with the experimental data, as increasing the temperature would be expected to disrupt aggregation and lead to sharper spectral resolution—whereas in certain solvents, the opposite trend is observed. Taken together, these findings suggest that the most plausible explanation for the observed line broadening is the presence of unpaired electrons, possibly due to thermal population of a low‐lying triplet state.

**Figure 3:**
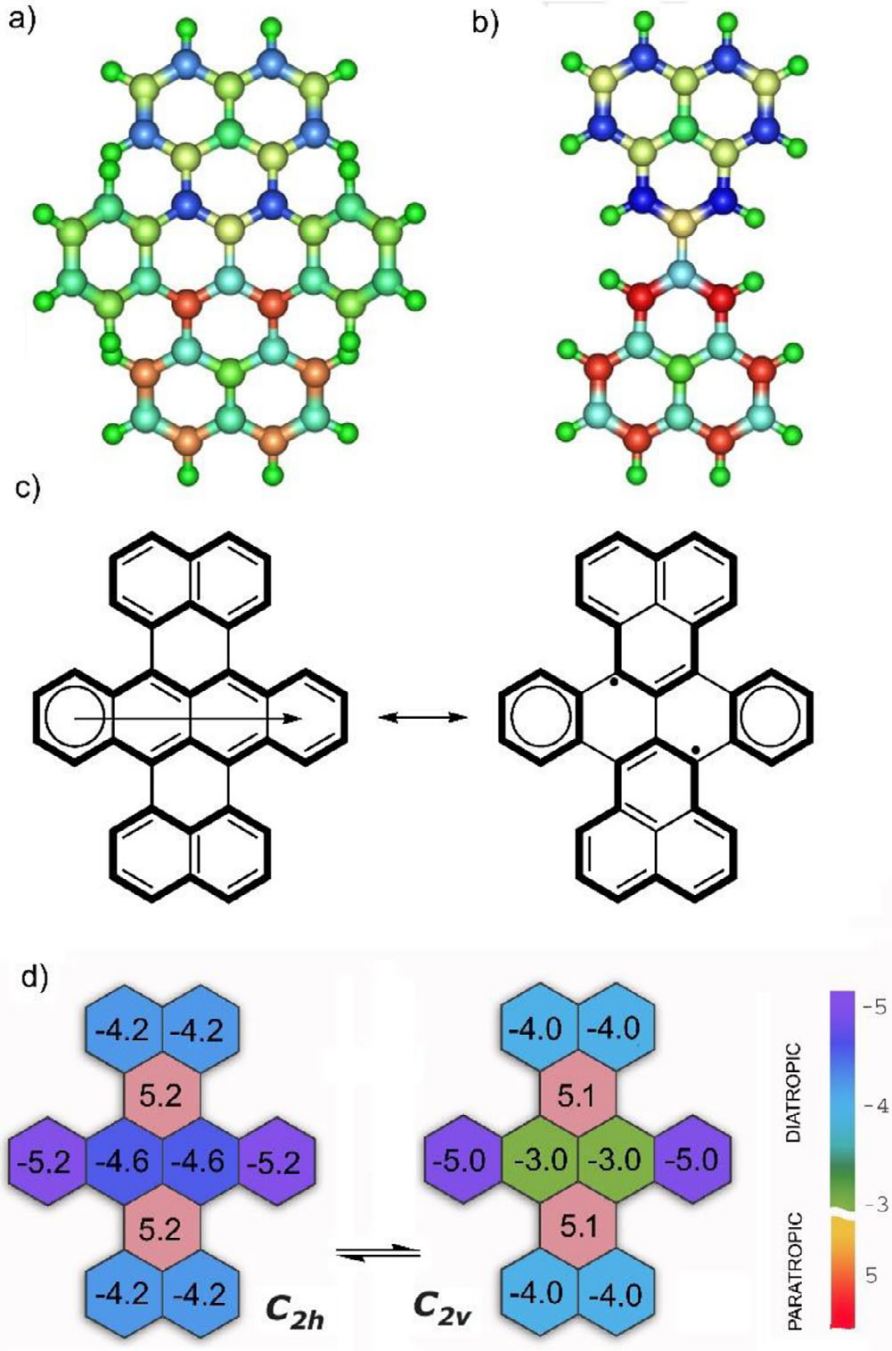
Mulliken spin densities on atoms for a) the *C*_2v_ conformer of DBT and b) unsubstituted biphenalenyl, highlighting the similarity in spin distribution between the two systems (colour range −0.35…0.35 for both cases), c) two key resonance structures of DBT: the left representing the closed‐shell form, and the right depicting the biradical form, d) NICS(0) analysis illustrating large differences in the ring current patterns in the two most stable DBT conformers, *C*_2h_ and *C*_2v_.

In light of this, the electronic structure of all identified DBT conformers was investigated in greater detail using DFT calculations. Spin density analysis from unrestricted DFT calculations reveals a clear biphenalenyl motif, interconnected by two o‐phenylene linkers (Figures 3a and 3b). The parent, unsubstituted biphenalenyl, is a well‐known disjoint biradical that has attracted significant attention in the literature due to its unusual electronic properties.^[^ ^26^, ^27^, ^28^, ^29^, ^30^, ^31^ ^]^ The strong similarity between the electronic structure of DBT and that of pristine biphenalenyl can be rationalized using Clar's sextet theory. As illustrated in Figure 3c, the biradical resonance forms gain an additional Clar sextet, which significantly stabilizes these structures and enhances their contribution to the overall electronic ground state. This additional aromatic stabilization makes the biradical form a major resonance contributor, despite the presence of extended conjugation through the o‐phenylene linkers.

However, in the case of DBT, the contribution of the closed‐shell resonance structure suppresses the biradical character through extended π‐conjugation across the two additional o‐phenylene linkers, thereby imparting enhanced molecular stability. Remarkably, the extent of this conjugation—which governs the quenching of biradical character—and the resulting balance between the closed‐shell and biradical mesomeric forms (Figure 3) differ significantly between the two most stable conformers, *C*_2h_ and *C*_2v_.

This can be illustrated qualitatively through bond length analysis. Notably, the bonds connecting the o‐phenylene linkers to the biphenalenyl core are significantly elongated in the *C*_2v_ conformer compared to *C*_2h_ (1.434 Å vs. 1.420 Å), indicating weaker conjugation between the fragments. This suggests that the Clar sextets in *C*_2v_ are more localized and isolated, whereas in *C*_2h_, they are better delocalized through the π‐system. Additionally, the *C*_2v_ conformer exhibits greater bond length equalization within the o‐phenylene fragments compared to *C*_2h_, a feature often associated with aromatic stabilization. Taken together, these observations support a greater contribution of the biradical mesomeric form in the electronic structure of the *C*_2v_ isomer.

Additionally, NICS(0) analysis confirms the presence of a strong paratropic ring current at the center of the DBT molecule, indicative of antiaromatic character in this region. The analysis also reveals more pronounced localization of Clar sextets within the o‐phenylene fragments in the *C*_2v_ conformer, further supporting its enhanced biradical character and reduced global conjugation compared to the *C*_2h_ isomer (Figure 3d).

Finally, we carried out a quantitative evaluation of the biradical character for both conformers. It is important to note that multiple computational protocols exist for estimating biradical character, and absolute values are meaningful only within the same methodological framework (see SI for details). In this work, we employed the Nakano biradical index, calculated from the natural orbital occupation numbers (HONO/LUNO) obtained at the PBE0 level of theory. This method provides results consistent with more advanced approaches, such as the configuration weights from CASSCF calculations.^[^ ^32^, ^33^, ^34^ ^]^ Using this approach, we obtained a biradical index of y = 0.01 for the *C*_2h_ conformer and y = 0.11 for the *C*_2v_ conformer. These values indicate that *C*_2h_ can be regarded as essentially closed‐shell, while *C*_2v_ exhibits clear biradicaloid character. To the best of our knowledge, such a striking difference in biradical character between two closely related conformers has not been previously discussed in the literature.

Systems with a biradical character comparable to or even lower than that of the *C*_2v_ conformer of DBT are often chemically unstable and tend to undergo dimerization, as exemplified by compounds such as sigmarene.^[^ ^35^ ^]^ Therefore, rather than being surprised by the low stability of DBT, one should instead ask why it is stable enough to persist at all. We assume that unusual radical localization in DBT is the reason for enhanced stability. Unlike many classical biradicals such as Chichibabin's or Thiele's hydrocarbon, where the unpaired electrons are localized on the pheriphery of the π‐ system, DBT exhibits a fundamentally different distribution. In this case, both the odd‐electron density (OED) and fractional occupation density (FOD) are highly localized on four central carbon atoms within the molecular core (Figure S17). This localization is further supported by the spin density map (Figure 3a and b), which shows significantly higher spin density on these central atoms compared to the peripheral regions. That can also be seen as 2,2'‐diallyl radical (tetramethylene‐ethane, TME) substituted with phenylene and naphthylene units.^[^ ^36^ ^]^ From a reactivity standpoint, the central carbon atoms are expected to be sterically less accessible to incoming reactive species and are partially shielded from both sides of the molecular plane by the surrounding helical moiety, which provides additional kinetic stabilization.

However, a high biradical character alone is not sufficient to account for the observed broadening in NMR spectra. In the absence of chemical reactions or photoexcitation, the only plausible source of unpaired electrons in a nominally closed‐shell organic molecule is thermal population of a low‐lying triplet state. If the singlet–triplet energy gap (ΔE_ST) is sufficiently small—typically less than 8–10 kcal/mol—the triplet state can be significantly populated at room temperature, leading to paramagnetic relaxation effects and resulting in NMR line broadening.^[^ ^37^ ^]^

**Figure 4:**
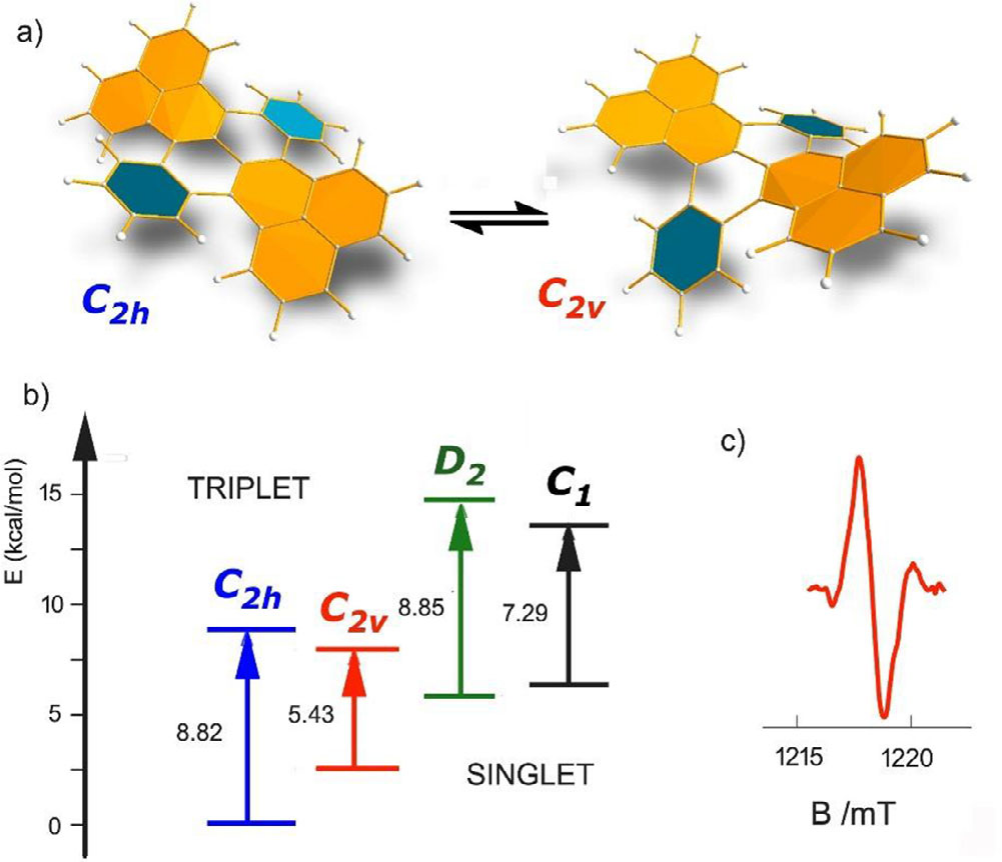
a) Schematic representation of the two main conformers *C*_2h_ and *C*_2v_; b) Calculated (DFT) singlet–triplet energy gaps (ΔE_ST) for DBT conformers; c) EPR measurements of DBT in o‐DCB solution (∼1219 mT, g ≈ 1.994).

This hypothesis is further supported by the observation that NMR signal broadening occurs in chlorinated solvents, while it is absent in hydrocarbon solvents. This behavior can be explained by the heavy‐atom effect, where halogenated solvents enhance intersystem crossing, thereby increasing the population of the paramagnetic triplet state and reducing spectral resolution. To corroborate this assumption, singlet–triplet energy gaps (ΔE_ST) were calculated for all DBT conformers. DFT simulations revealed that the *C*_2v_ conformer has a very small singlet–triplet gap, at 5.4 kcal/mol, and its triplet state is also lower in energy than that of the *C*_2h_ conformer (Figure 4). While it is important to note that DFT‐based energy gaps can sometimes underestimate ΔE_ST, such a small value makes thermal population of the triplet state highly plausible, particularly in the case of the *C*_2v_ conformer.

To directly probe the presence of a thermally populated triplet state in DBT, EPR measurements were performed. A weak but clearly distinguishable EPR signal was observed in o‐DCB solution (Figure 4c; ∼1219 mT, g ≈ 1.994), while no deviation from background was detected in DCM or benzene solutions. Furthermore, temperature‐dependent EPR measurements in o‐DCB (Figure S12) revealed that the distinctive signal of DBT was observed only at 30 °C, consistent with the thermal population of a paramagnetic species. Upon further heating to 50 °C, the signal intensity decreased, accompanied by sample decomposition. These results align well with the NMR observations, supporting the interpretation that the triplet state—presumably associated with the *C*_2v_ conformer—is thermally accessible, with solvent‐dependent triplet population following the trend: benzene < DCM < *o*‐DCB.

## 3 Conclusions

In this work, we performed a comprehensive characterization of DBT. Despite its low solubility and limited stability, we successfully conducted, for the first time, high‐quality NMR analysis, PXRD, and EPR spectroscopy on this fundamental polycyclic aromatic hydrocarbon. We demonstrate that DBT can be viewed as a Clar‐sextet‐interlocked biphenalenyl system, featuring unusual localization of radical centers at the molecular core—an arrangement that likely contributes to its unexpected persistence despite the inherent reactivity of biradicaloids. These findings highlight DBT as a promising candidate for future applications and the design of functional organic materials with controllable open‐shell character. Combined with an extensive DFT study, our results reveal a unique case in which a conformational switch transforms a molecule from an essentially closed‐shell system to one with a significantly pronounced biradical character. This geometry‐induced control over electronic ground states opens promising avenues for the design of stable, low‐gap open‐shell systems, which are of considerable interest for molecular spintronics, quantum information science, and responsive organic materials.

## Supporting Information

The authors have cited additional references within the Supporting Information.

## Conflicts of Interest

The auhtors declare no conflicts of interest.

## Supporting information



Supporting Information

## Data Availability

The data that support the findings of this study are available in the supplementary material of this article.
