# Supplementary material for: The Mystery of Dibenzoterrylene: A Clar‐interlocked Biphenalenyl Biradical
Source: Chemistry. 2025 Nov 10;31(71):e02952. doi: 10.1002/chem.202502952 (PMC12734663; doi:10.1002/chem.202502952)
Supplement: Supplementary file 1 — Supporting Information [file CHEM-31-e02952-s001.pdf]

The Mystery of Dibenzoterrylene: A Clar-interlocked Biphenalenyl Biradical

Elena A. Kalinina, [a,b] Mikhail A. Kalinin,[b] Dmitry I. Sharapa,[c] Harald Maid,[d] Alexandra Freidzon,[e] Haleh Hashemi Haeri,[f] Dariush Hinderberger,[f] Robert Dinnebier,[g] Alexander S. Oshchepkov,[a,b*] Konstantin Yu. Amsharov*[b]

[a.] *Max Planck Institute for the Science of Light, Department of Physics, D-91058 Erlangen, Germany. E-mail: aleksandr.oshchepkov@mpl.mpg.de*

[b.] *Institute of Chemistry, Organic Chemistry, Martin-Luther-University Halle-Wittenberg, 06120 Halle, Germany. E-mail: konstantin.amsharov@chemie.uni-halle.de*

[c.] *Institute of Catalysis Research and Technology, Karlsruhe Institute of Technology (KIT), Herrmann-von-Helmholtz-Platz 1, 76344 Eggenstein-Leopoldshafen Germany*

[d.] *Institute of Organic Chemistry II, University Erlangen-Nuremberg, Nikolaus-Fiebiger-Str. 10, Erlangen 91058, Germany*

[e.] *Department of Molecular Chemistry and Materials Science, Weizmann Institute of Science, Rehovoth, 7610001, Israel*

[f.] *Institute of Chemistry, Physical Chemistry – Complex Self-Organizing Systems, Martin Luther University Halle-Wittenberg, 06120 Halle (Saale), Germany*

[g.] *Max Planck Institute for Solid State Research, 70569 Stuttgart Germany*

Table of contents

Instrumental Parameters 2

Synthesis 3

Characterisation (NMR spectra; UV; HPLC) 5

EPR 14

Measurement of the stability 15

Computational details 21

XYZ coordinates 23

References 30

Instrumental Parameters

All chemicals and solvents were purchased in reagent grade from commercial suppliers (Acros®, Sigma-Aldrich® or Fluka®, Fluorochem®, Merck®, ChemPur®) and used as received, unless otherwise specified. Solvents in HPLC grade were purchased from VWR® and Sigma-Aldrich®.
Flash column chromatography was performed on an Interchim PuriFlash XS420 using flash grade silica gel from (Machery-Nagel 60 M (40–63 mm, deactivated)).
NMR spectra were recorded on a Bruker Avance 400 at 400 MHz (^{1}H NMR) and 100 MHz (^{13}C NMR). The signals were referenced to residual solvent peaks (in parts per million (ppm) ^{1}H: $CDCl_3$, 7.27 ppm; CD_2Cl_2, 5.32 ppm; C_6D_6 7.16 ppm; $C_6D_4Cl_2$ 6.93 ppm, 7.19 ppm; ^{13}C: $CDCl_3$, 77.0 ppm; CD_2Cl_2, 53.84 ppm; C_6D_6 128.39 ppm. Coupling constants were assigned as observed. The obtained spectra were evaluated with the program ACDlabs.
Proton spectra of DBT (**4**) in C_6D_6, CD_2Cl_2 and $C_6D_4Cl_2$ were recorded on a Bruker 600 MHz Avance NEO operating at 600,65 MHz equipped with a cryoprobe (CPDCH600S3 C/H-D-05Z). 32 transients with 128k time domain data points were recorded, one time zerofilled and processed using a line broadening of 0.3 HZ. To yield the correct coupling constants, the triplet at 7.76 ppm was deconvoluted using the dcon routine implemented in TOPSPIN 4.4.1.Proton.
(MA)LDI-MS spectra were recorded on a Shimadzu Biotech AXIMA Confidence MALDI-TOF. DCTB matrix (trans-2-[3-(4-tert-Butylphenyl)-2-methyl-2-propenylidene]malononitrile)
HR-MS measurements Thermo Scientific Q Exactive Plus spectrometer equipped with an Orbitrap Mass Analyzer ion source LIFDI (liquid injection field desorption ionization) by Linden CMS, Germany
HPLC measurements were performed on a Shimadzu Prominence Liquid Chromatograph LC-20AT with communication bus module CBM-20A, diode array detector SPDM20A, the degassing unit DGU-20A5 R, column oven CTO-20AC or CTO-20A, respectively and with auto sampler SIL- 20A HT. For separation a Cosmosil 5-PBR column (4.6 mm x 250 mm) from Nacalai Tesque was used, as eluent a toluene/MeOH was used. The data was evaluated with the programs Shimadzu LC solution and Shimadzu LabSolutions.
Absorption spectra were measured in 1 cm quartz cuvettes with Shimadzu UV-2600I Spectrometer.
Fluorescence spectra were recorded on a Shimadzu RF-6000 spectrofluorophotometer. The samples were measured in quartz cuvettes at room temperature.
EPR X-Band (9.43 GHz) CW-EPR spectra were acquired using a MiniScope MS400 benchtop spectrometer (Magnettech, now Bruker BioSpin). Spectra were recorded with microwave power of 10 mW, 100 KHz modulation frequency and modulation amplitude of 0.3 mT and 4096 points. The MATLAB based EasySpin software package (version 6.0.5) was used for spectral simulations [1]. The natural abundancy of nuclei was considered through simulations.

Synthesis

The synthetic route is shown in Scheme 1. The basic synthetic pathway was developed based on the method proposed by Clar in 1955[2].

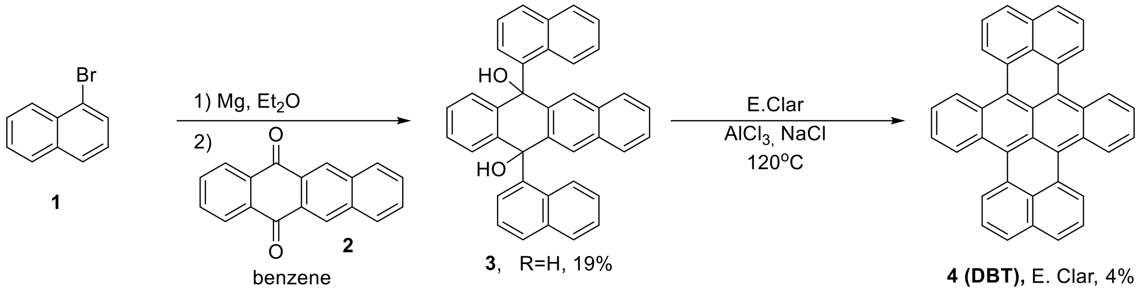

Scheme S1. The main synthetic route toward DBT.

5,12-di(naphthalen-1-yl)-5,12-dihydrotetracene-5,12-diol (3)

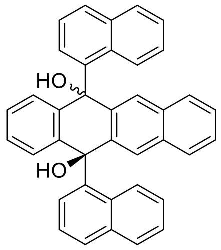

1-bromo naphthalene (6.76 ml, 48.3 mmol) were added to 50 ml of dry ether, cooled to -78°C in an isopropanol bath and 21.25 ml (2.5 M, 53.1 mmol) of BuLi were added. A white precipitate formed, suspension was stirred for 1 hour. The reaction mixture was warmed to 0°C and a suspension of tetracene-5,12-dione (3.12 g, 12.1 mmol) in 100 ml of dry benzene was added by portions. The reaction mixture turned yellow, then green and turned black. The mixture was stirred with boiling overnight. Reaction was cooled to room temperature, 50 ml of ethanol and 50 ml of water were added. The reaction mixture turned violet, then green, and then dark pink and white precipitate formed. The precipitate was filtered, yielding 0.92 g of a white powder (trans isomer). The solution was evaporated and purified by column chromatography on silica gel. Eluent hexane:DCM = 3:7. In this system, it was possible to separate the trans isomer (133 mg) and the cis isomer (97 mg). A crystal was obtained from the cis isomer by vapor transfer in the chloroform:pentane system. The overall yield was 19%. Only the trans isomer was used in the following reactions.

Cis: ^{1}**H NMR** (500 MHz, $CDCl_3$) δ 2.75 (s, 2 H) 7.04 - 7.14 (m, 4 H) 7.22 (dd, J=5.87, 3.42 Hz, 2 H) 7.25 - 7.29 (m, 4 H) 7.49 - 7.56 (m, 2 H) 7.71 (s, 2 H) 7.78 - 7.85 (m, 4 H) 7.98 (d, J=8.07 Hz, 2 H) 8.09 (d, J=8.56 Hz, 2 H) 8.81 (dd, J=7.21, 1.10 Hz, 2 H)

^{13}**C NMR** (126 MHz, $CDCl_3$) δ 75.19; 124.50; 124.63; 125.14; 125.59; 126.29; 126.90; 127.40; 127.68; 127.86; 128.49; 129.16; 129.18; 129.84; 133.35; 134.66; 137.97; 139.00; 142.49.

Trans: ^{1}**H NMR** (500 MHz, CDCl3) δ 3.95 (s, 1 H) 3.96 (s, 1 H) 6.82 (dd, J=5.87, 3.18 Hz, 2 H) 6.99 - 7.02 (m, 2 H) 7.04 (td, J=7.76, 1.10 Hz, 2 H) 7.15 (d, J=8.80 Hz, 2 H) 7.23 - 7.26 (m, 2 H) 7.31 (s, 2 H) 7.32 - 7.36 (m, 2 H) 7.38 - 7.43 (m, 2 H) 7.84 (dd, J=8.19, 7.46 Hz, 2 H) 7.96 (d, J=8.07 Hz, 2 H) 8.08 (d, J=8.07 Hz, 2 H) 8.52 - 8.60 (m, 2 H)

HRMS: Chemical Formula: $C_{38}H_{26}O_2$ calc. 514.1933, found 514.1930.

Synthesis of DBT

5,12-di(naphthalen-1-yl)-5,12-dihydrotetracene-5,12-diol (1g, 1,94 mmol) was added to the melt of aluminum chloride (10 g, 75 mmol) and sodium chloride (2g, 34 mmol) in argon atmosphere at 150 degrees. Brown mass was stirred in an oil bath for 10 minutes. Then mixture was cooled to room temperature then 20 ml 10% hydrochloric acid was added. The resulting dark green mixture was boiled for another 10 minutes. Then the reaction mixture was transferred to a beaker, where hydrochloric acid was neutralized with a solution of sodium carbonate. The resulting green precipitate was filtered and washed with water, 20 ml of ethanol and dried on air. The green precipitate (780 mg) was boiled in 20 ml p-xylene and hot mixture was filtered. Procedure was repeated 3 times. The resulting precipitate is DBT (256 mg, 26%) with purity 93%. After high-vacuum sublimation (200°C, $1*10^{-7}$ mbar) DBT was obtained with 4% yields.

Tribenzo[de,h,kl]naphtho[1,2,3,4-rst]pentaphene (DBT); (4)

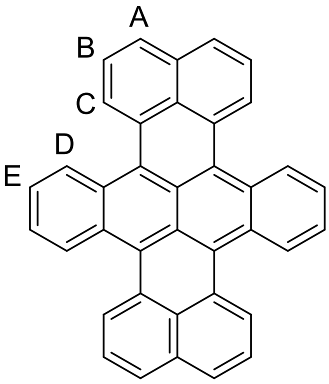

Dark green solid (39 mg, 4%).

^{1}H NMR (600.65 MHz, CD_2Cl_2, 25 °C) δ 7.49 - 7.55 (m, 4 C_EH) 7.76 (dd, ^3J = 7.5 Hz, ^3J = 8.0 Hz, 4 C_BH) 7.96 (d, ^3J = 8.0 Hz, 4 C_AH) 8.41 (d, ^3J = 7.5 Hz, 4 C_CH) 8.77 - 8.83 (m, 4 C_DH)
^{1}H NMR (500.34 MHz, $C_{10}D_8$, 100 °C) δ 7.38 - 7.43 (m, 4 H) 7.50 (t, ^3J = 7.8 Hz 4 H) 7.70 (d, J=8.1 Hz, 4 H) 8.49 (d, J=7.5 Hz, 4 H) 8.95 - 9.02 (m, 4 H)
MS (LDI): *m/z (real. int.)* = 476.10 $[M]^+$ (100)
UV/Vis (Toluene, 293 K): λ [nm]) = 742, 678, 373, 346, 320, 285.

Characterisation (NMR spectra; UV; HPLC)

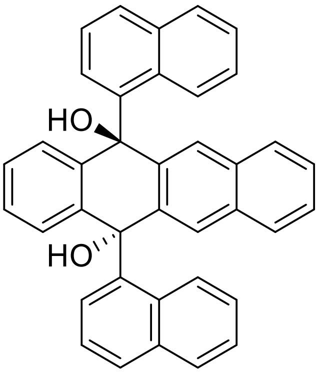

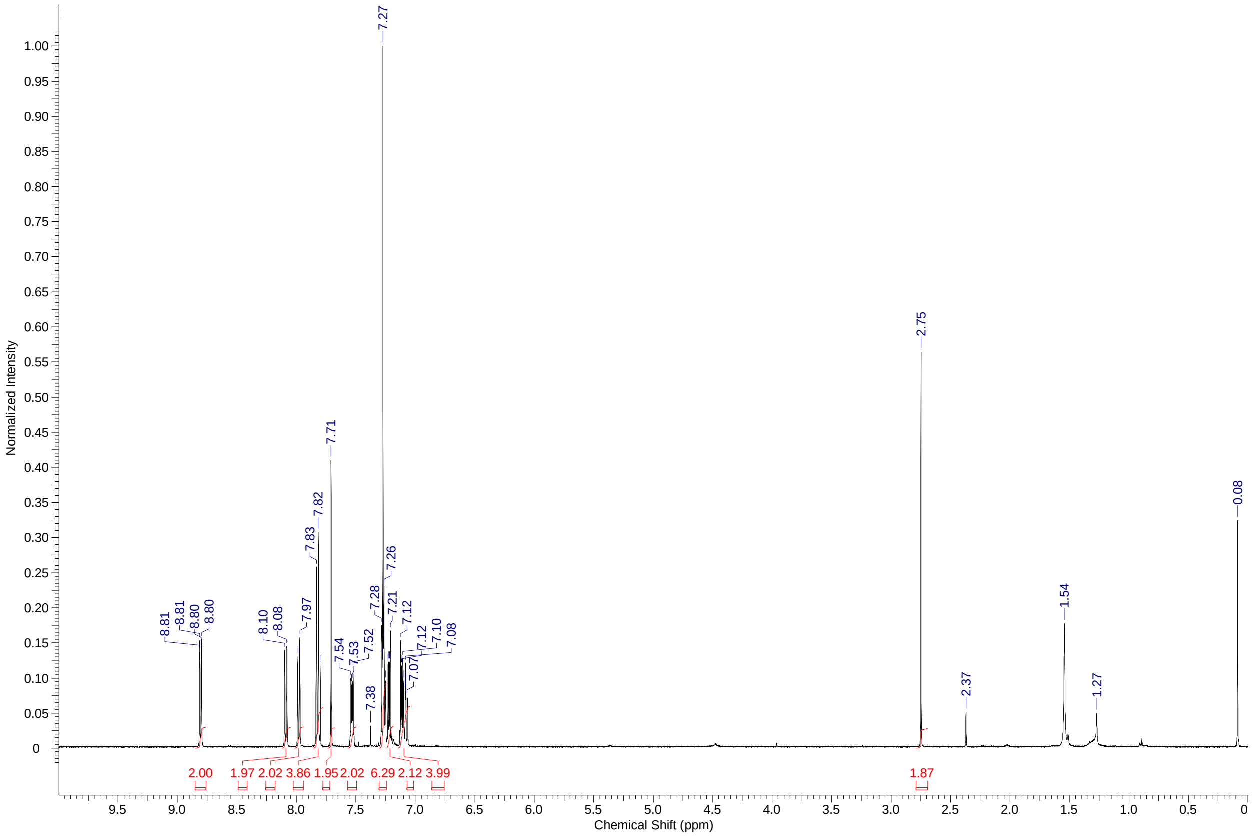

Figure S1. ^{1}H NMR (400 MHz, $CDCl_3$, 293 K) (5R,12R)-5,12-di(naphthalen-1-yl)-5,12-dihydrotetracene-5,12-diol (3)

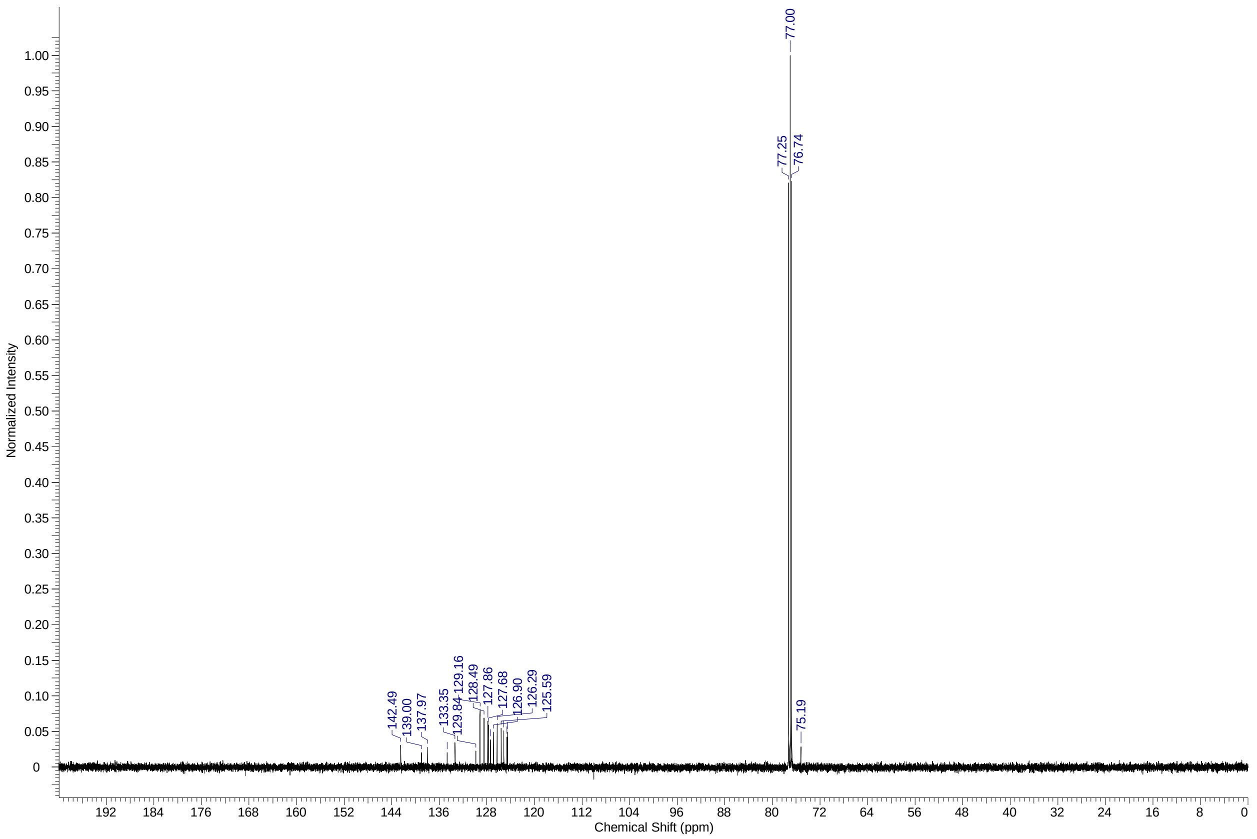

Figure S2. ^{13}C NMR (101 MHz, $CDCl_3$, 293 K) spectrum of (5R,12R)-5,12-di(naphthalen-1-yl)-5,12-dihydrotetracene-5,12-diol (3)

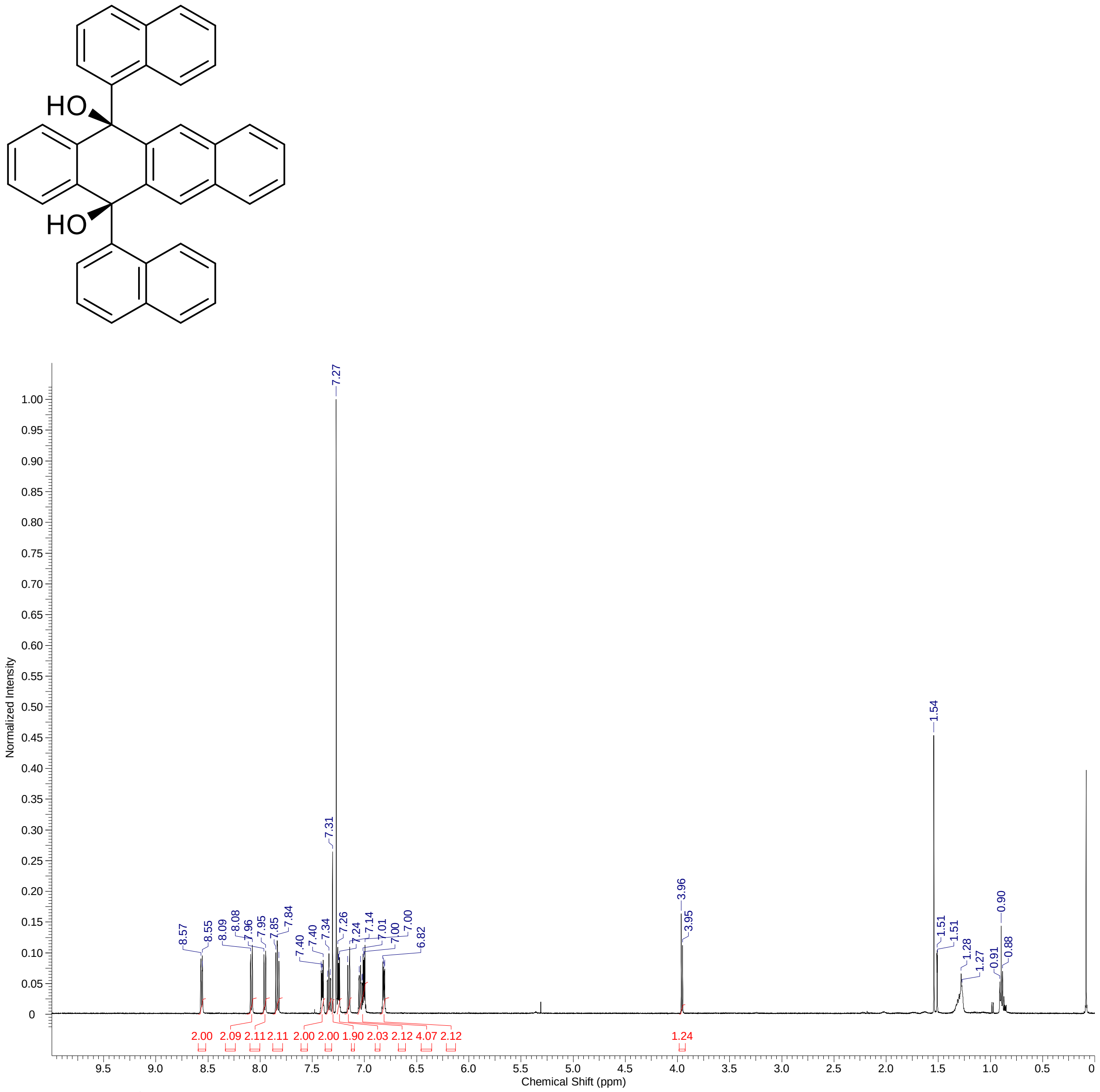

Figure S3. ^{1}H NMR (400 MHz, $CDCl_3$, 293 K) spectrum of (5R,12S)-5,12-di(naphthalen-1-yl)-5,12-dihydrotetracene-5,12-diol (3)

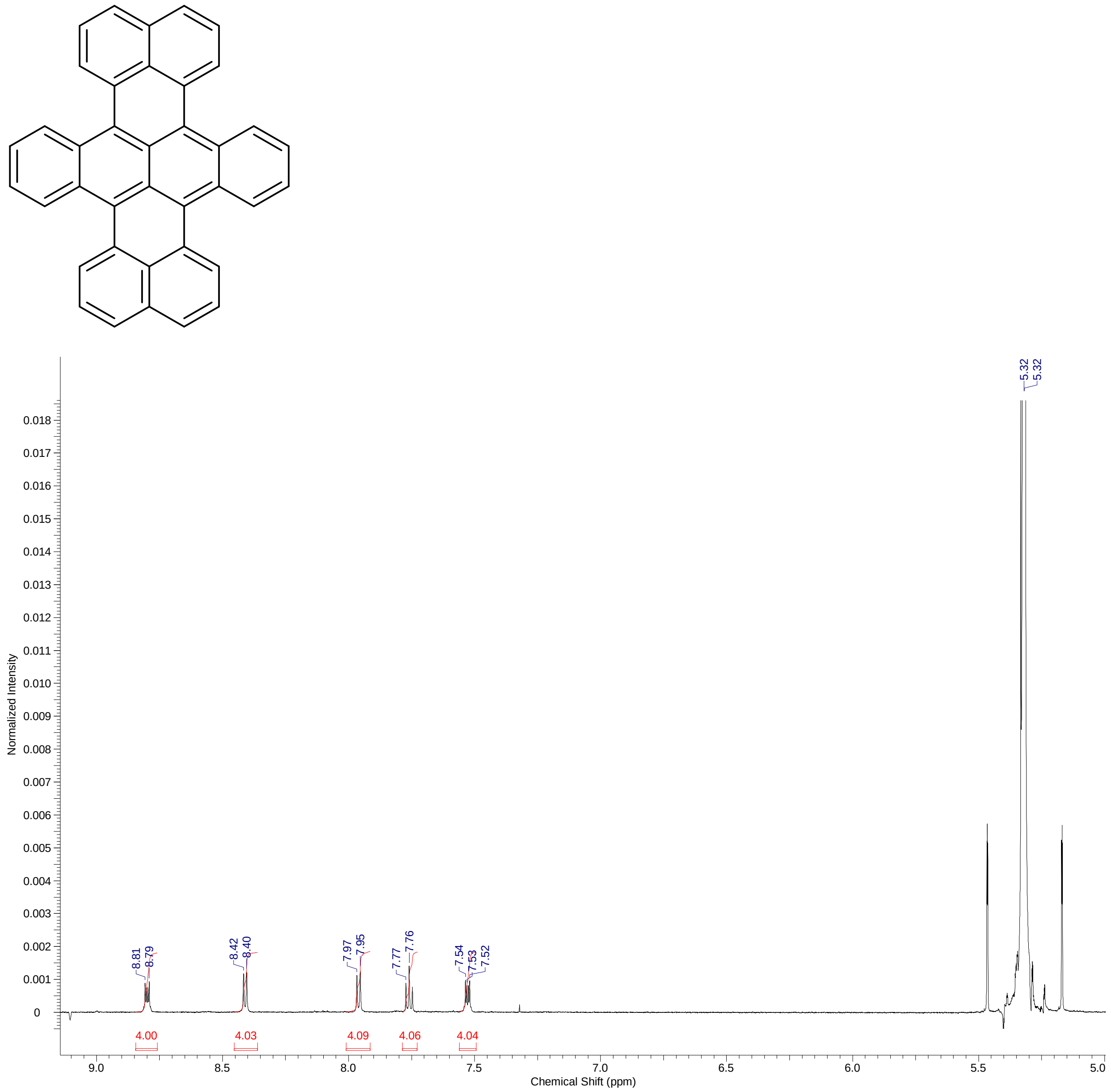

Figure S4. ^{1}H NMR (600 MHz, CD_2Cl_2, 298 K) spectrum of tribenzo[de,h,kl]naphtho[1,2,3,4-rst]pentaphene (DBT)

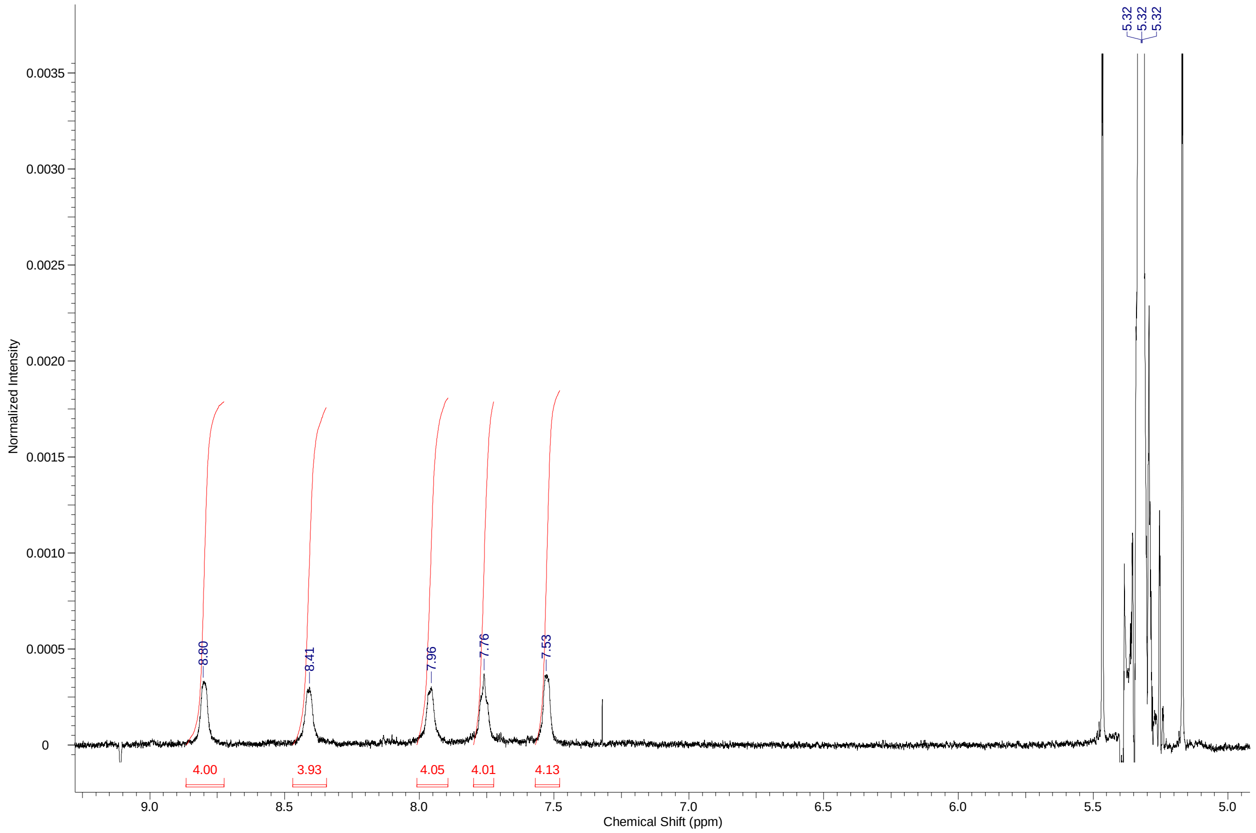

Figure S5. ^{1}H NMR (600 MHz, CD_2Cl_2, 298 K) spectrum of tribenzo[de,h,kl]naphtho[1,2,3,4-rst]pentaphene (DBT) 3h later

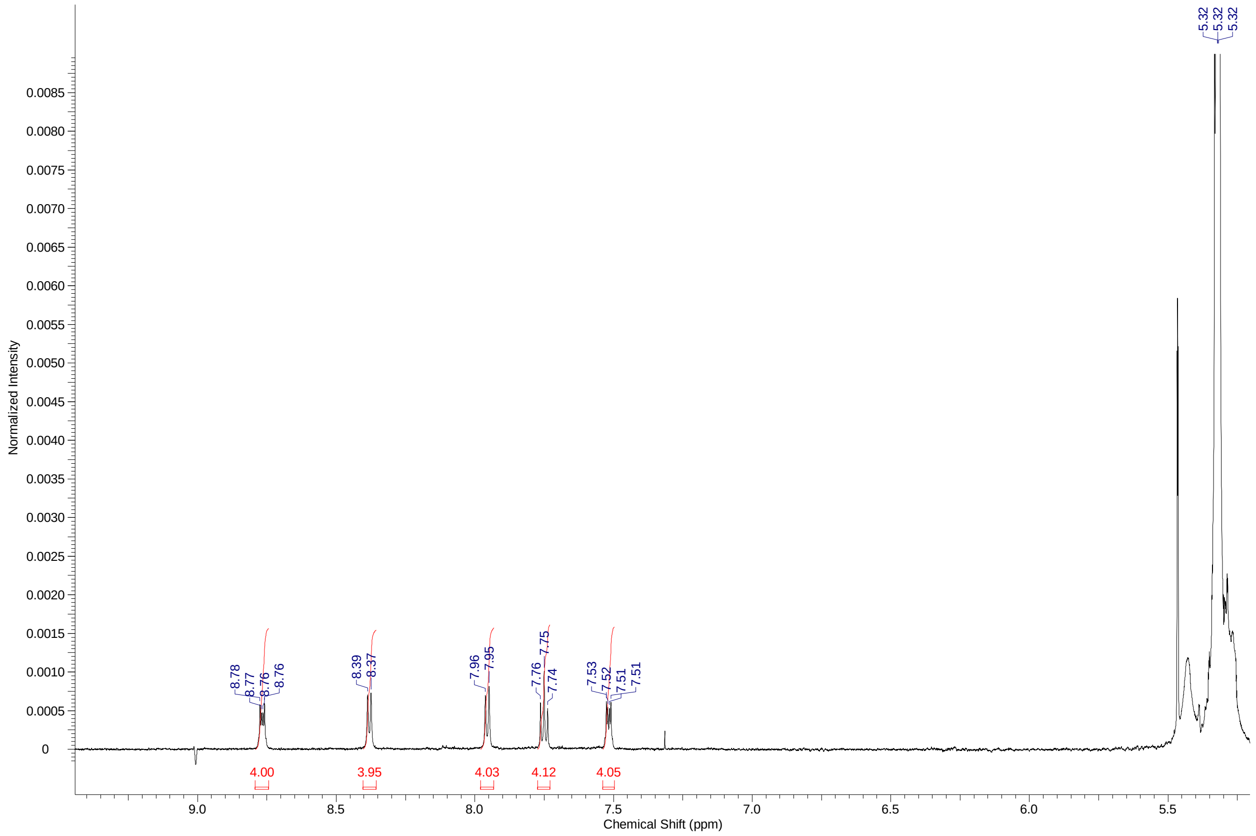

Figure S6. ^{1}H NMR (600 MHz, CD_2Cl_2, 253 K) spectrum of tribenzo[de,h,kl]naphtho[1,2,3,4-rst]pentaphene (DBT) 3h later

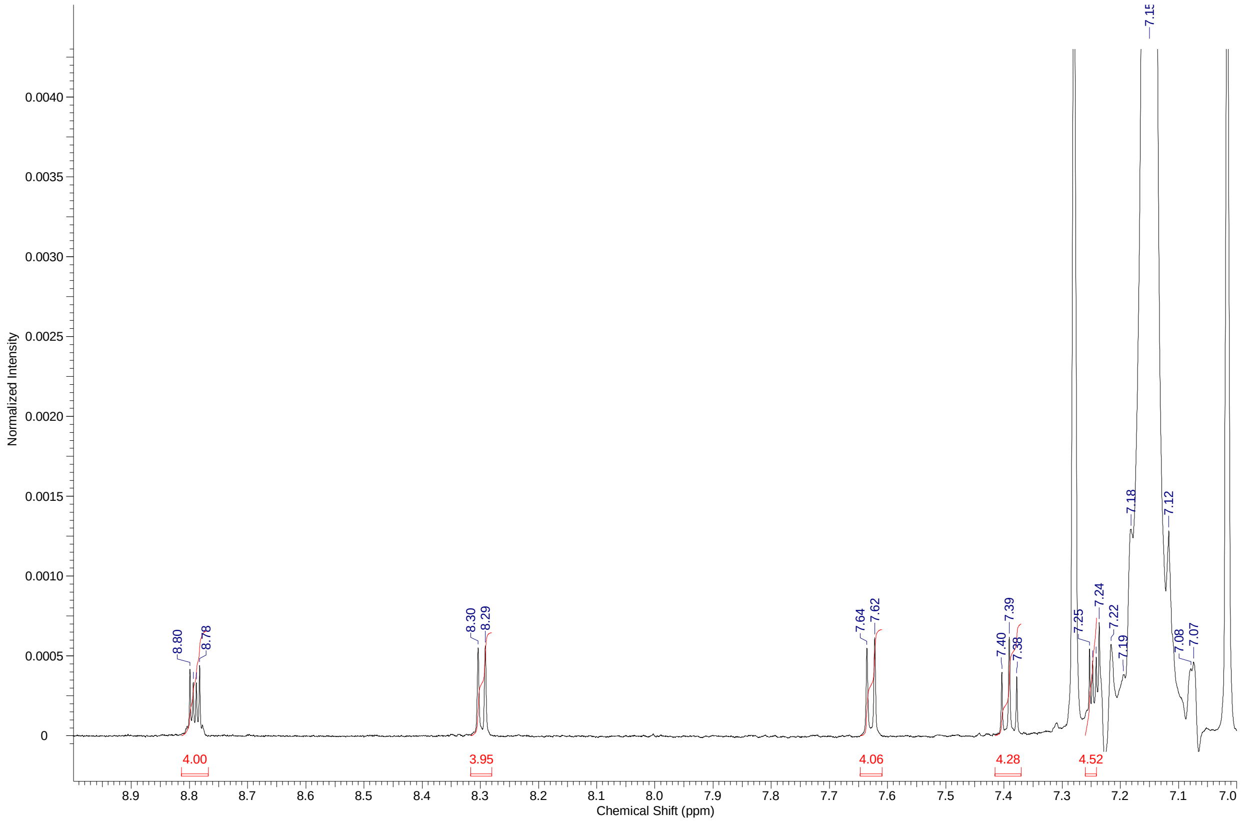

Figure S7. ^{1}H NMR (600 MHz, C_6D_6, 298 K) spectrum of tribenzo[de,h,kl]naphtho[1,2,3,4-rst]pentaphene (DBT)

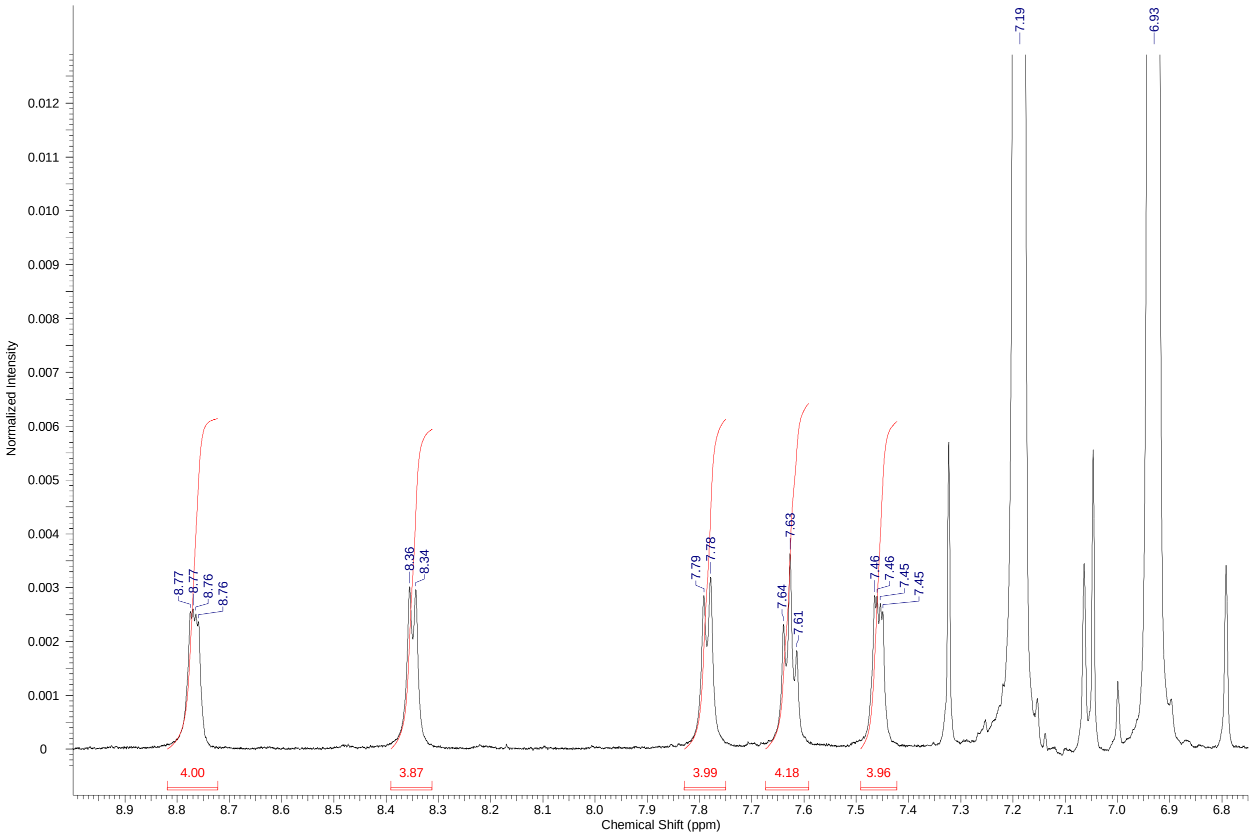

Figure S8. ^{1}H NMR (600 MHz, $C_6D_4Cl_2$, 298 K) spectrum of tribenzo[de,h,kl]naphtho[1,2,3,4-rst]pentaphene (DBT)

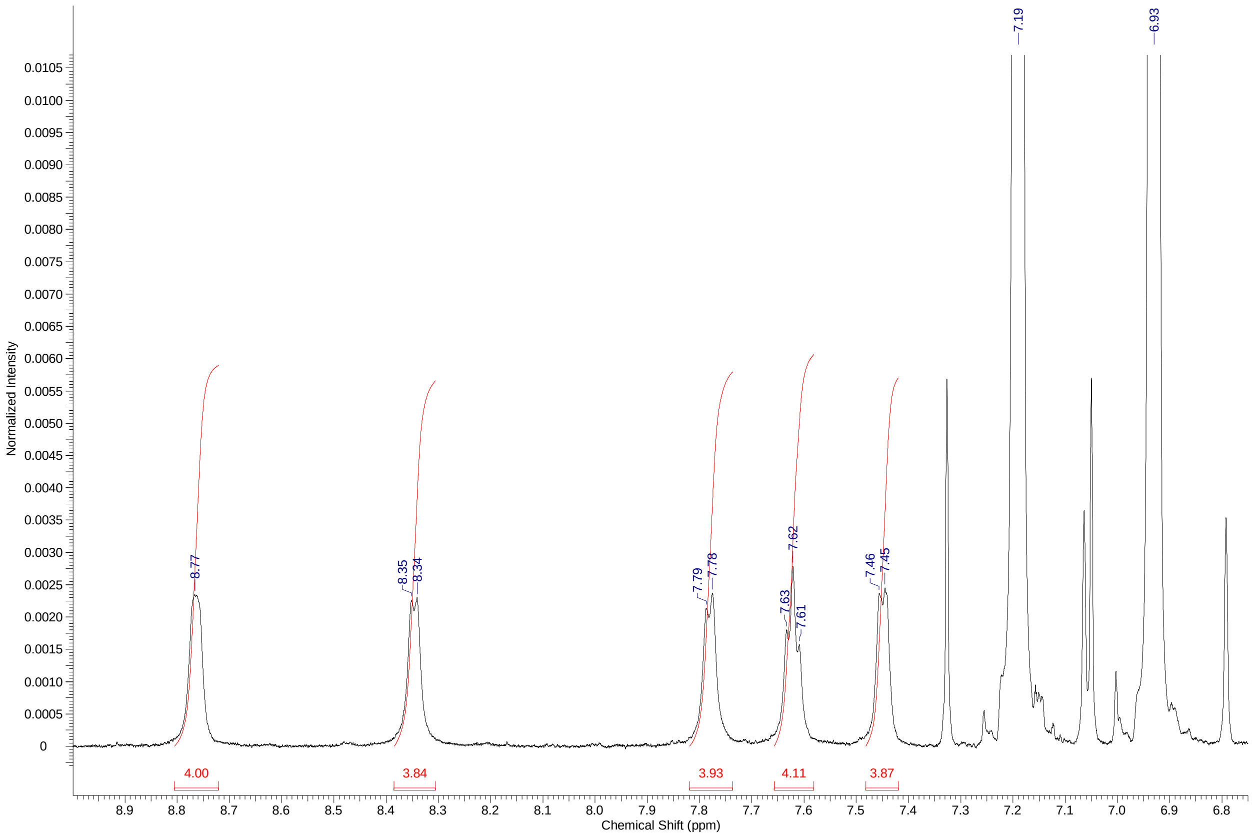

Figure S9. ^{1}H NMR (600 MHz, $C_6D_4Cl_2$, 338 K) spectrum of tribenzo[de,h,kl]naphtho[1,2,3,4-rst]pentaphene (DBT)

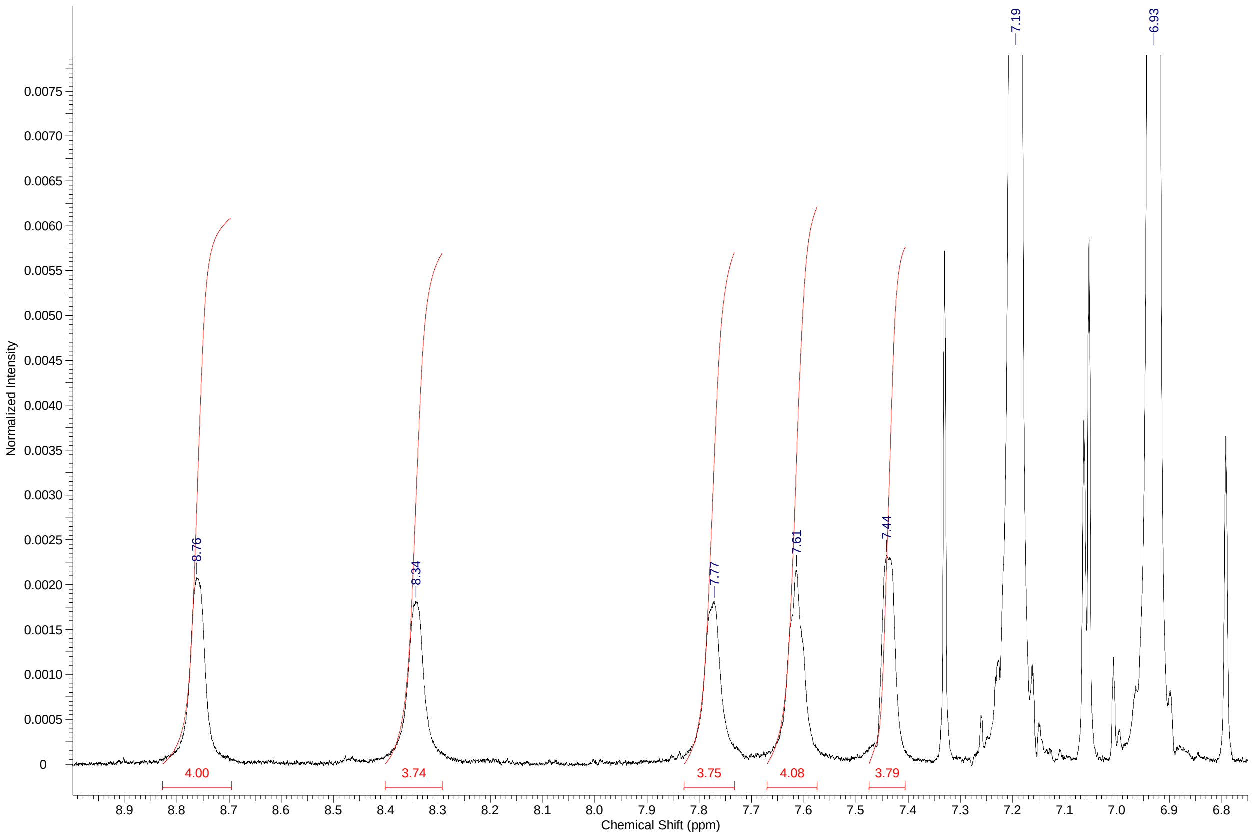

Figure S10. ^{1}H NMR (600 MHz, $C_6D_4Cl_2$, 358 K) spectrum of tribenzo[de,h,kl]naphtho[1,2,3,4-rst]pentaphene (DBT)

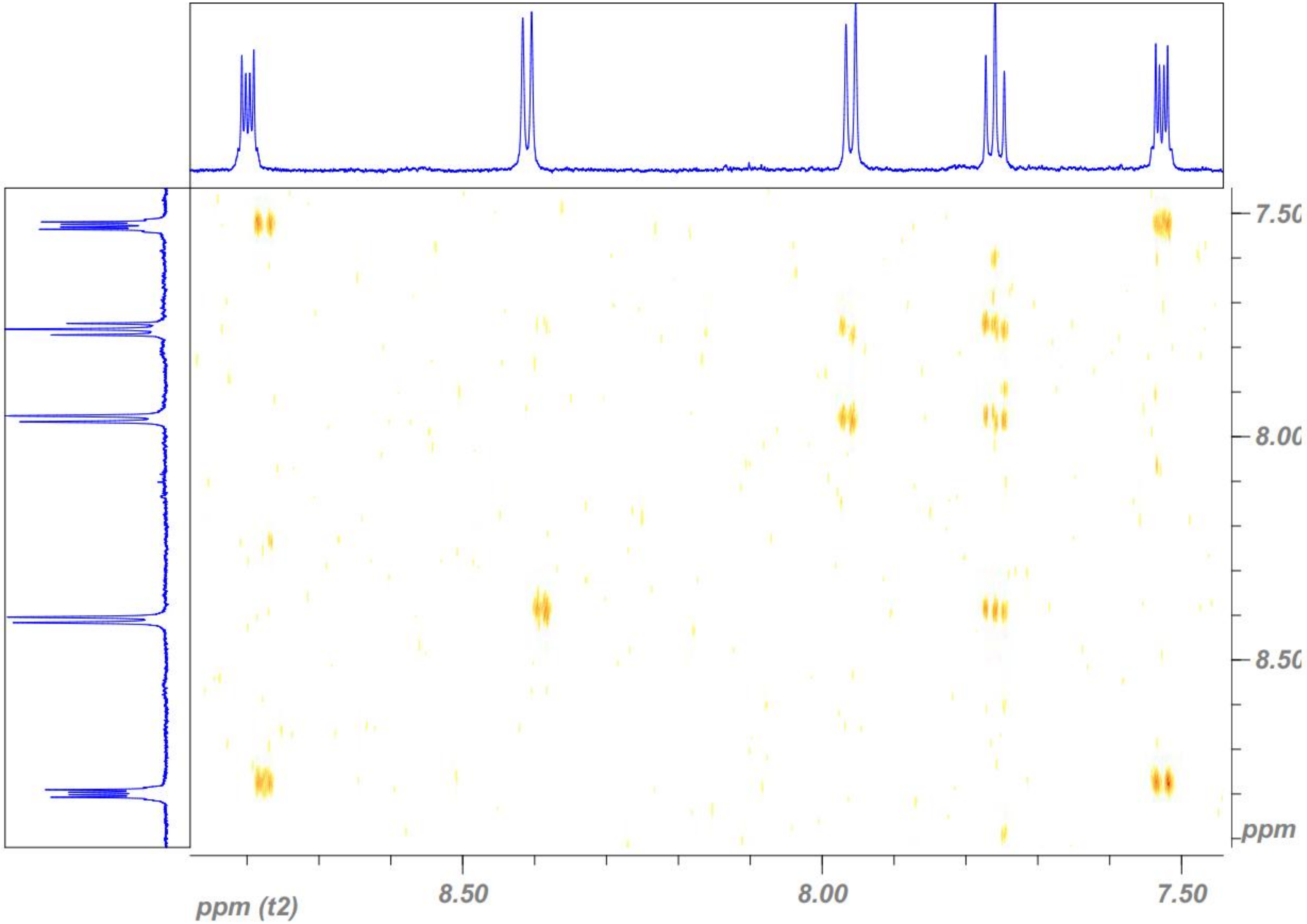

Figure S11. ^{1}H-^{1}H-COSY NMR spectrum of tribenzo[de,h,kl]naphtho[1,2,3,4-rst]pentaphene (DBT) in CD_2Cl_2.

EPR

The cw-EPR spectrum of DBT in o-DCB could be observed only at 30°C and no other temperatures. The obtained signal is displayed in Fig. S12. It is a broad Lorentzian lineshape (ΔBpp~5mT) with a giso value centered around 2.0, indicative a carbon centred radical. Spectral simulation revealed a rather axial g-tensor with g= [2.0052 2.0051 2.0015] and giso=2.0039. The isotropic g-value is in agreement with g- values found for carbon based radicals [3-5].

The broadening of the spectrum could be due to the delocalization of the electron through the dibenzoterrylene (DBT) structure. It could also be attributed to the presence of DBT species with slightly different g-values. Spectral simulation indeed was possible using g-strain option on perpendicular (gxx, gyy) direction. No Signal could be obtained for DBT dissolved in DCM or Benzene solutions.

As a measure of produced spins, doubly integrated signal was calculated, which resulted about 48% of the initial concentration of DBT in dissolved o-DCB (6.3×10^{-4} mol/lit).

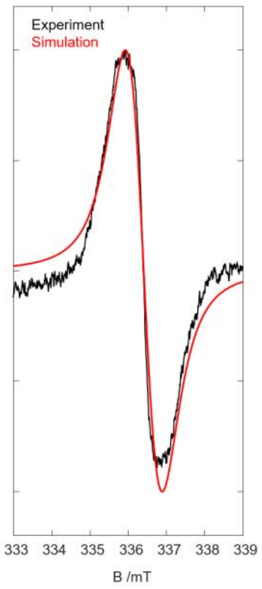

Figure S12. Experimental (in black) and Simulated (in red) spectra of DBT dissolved in *o*-DCB at 30°C

Measurement of the stability

Solution of DBT in toluene with a concentration of 1.5 x 10^{-5} M was prepared in 2 ml vials. The solution was stored at room temperature under laboratory lighting conditions. Spectra were taken every 24 hours.

The half-life of the DBT in solution was calculated using the following equation:

$$A=a-b\cdot c^{t}$$

Where *A* is the absorption at a given time *t* after the start of the experiment, and *a*, *b*, and *c* are variable coefficients.

The half-life of DBT in toluene in daylight and air is 39h.

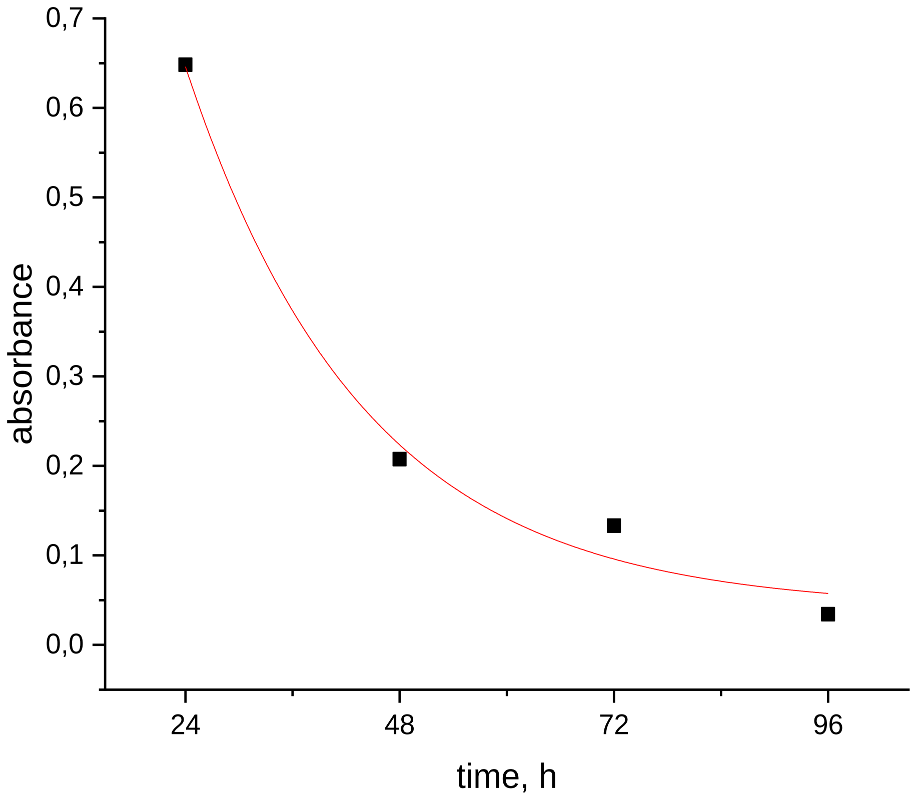

Figure S13. Stability graph of DBT in toluene in daylight and air with a concentration of 1.5 x 10^{-5} M.

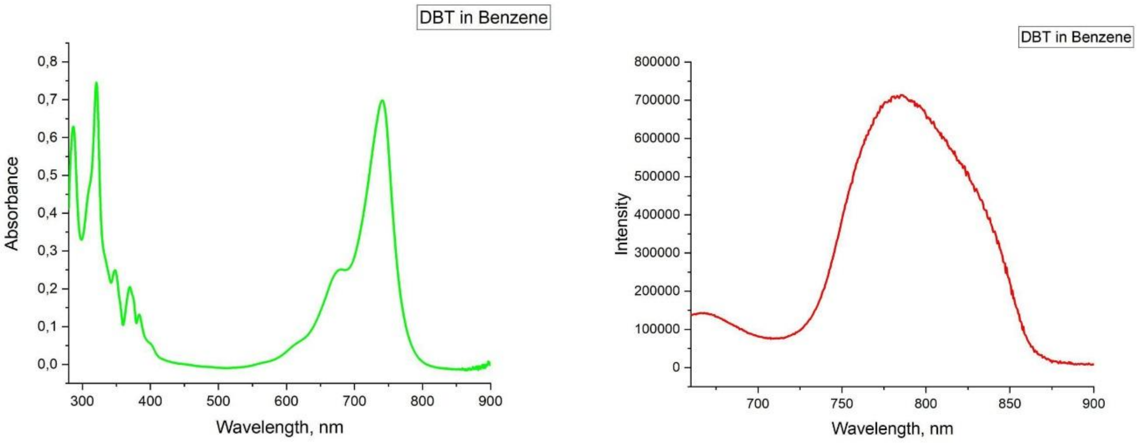

Figure S14. Absorption (left) and fluorescence (right) spectra of DBT solution in benzene ($C=1*10^{-5}$ M). Parameters: λ_{ex}= 650 nm, slits 5:5. Additional parameters, as well as raw data in xlsx format, are provided in the “Parameters” file.

The data correlates with the literature[6].

X-ray analysis

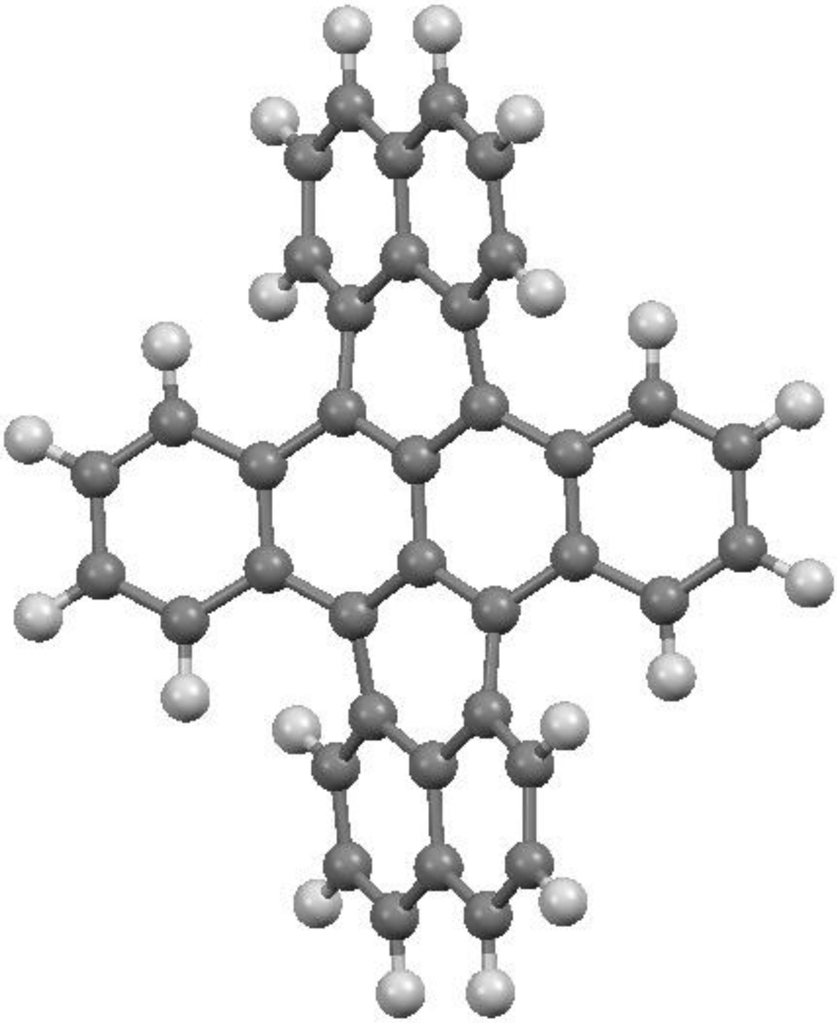

Figure S15. Molecular structure of DBT

Laboratory X-ray powder diffraction (XRPD)

X-Ray Powder Diffraction (XRPD) measurements for structure determination were performed by using a Stoe Transmission Powder Diffraction System (STADI-P) equipped with a Ge(111) Johann-type monochromator from STOE & CIE with Cu-Kα_1 radiation (λ = 1.54059 Å) that was equipped with an array of three linear position-sensitive MYTHEN 1K detectors from Dectris Ltd. of approximately 18° 2θ opening angle each. The finely powdered sample of Dibenzoterrylene (DBT) was placed in a glass capillary of 0.7 mm (Hilgenberg glass No. 14) and spun during measurement or improving particle statistics. The measurement in the range from 2.0 – 112° 2θ with a step width of 0.015° 2θ took 3 hrs.

For indexing of the powder pattern of DBT at T = 298 K, the program TOPAS version 7 [7] was used. Indexing was performed by iterative use of singular value decomposition (LSI) [8], leading to a primitive orthorhombic unit cell with unit cell parameters of a = 24.1912(6) Å, b = 12.1247(3), and c = 7.76541(7) (V = 2277.69(8) Å^3). The possible space group could be determined as centrosymmetric Pbcn from the observed extinction rules and was confirmed after structure

determination. The number of formula units per unit cell could be estimated to Z = 4 from volume increments or half a molecule in the asymmetric unit. The peak profiles and precise lattice parameters of the powder patterns of DBT were first determined by a Pawley fit [9] using the fundamental parameter (FP) approach of TOPAS [10]. Beforehand, the instrumental peak profile was determined using the NIST LaB6 SRM 660C line profile standard [11] applying the Thompson-Cox-Hastings Pseudo-Voigt function [12] using 4-line profile parameters. For the modelling of the background, Chebychev polynomials were employed. The apparent anisotropic with of the Bragg peaks was modelled by the phenomenological microstrain model of Stephens [13], revealing the directions of maximum and minimum "disorder" in the crystal structure of DBT (Fig. S16). Several very small reflections of an unknown impurity phase were modelled by single peak fitting. The refinement converged quickly.

Structure determination of DBT was performed in an iterative manner by the global optimization method of simulated annealing in real space using the TOPAS program [14]. The DBT molecule as a rigid body (half occupancy) in four possible molecular conformations from previous DFT calculations was subjected to the simulated annealing process with 6 external degrees of freedom, three translational and three rotational. Only the conformation (C_{2h}) with the lowest relative energy let to an excellent match with the powder pattern. Unsurprisingly the centre of mass of the molecule located on a centre of symmetry at the origin of the unit cell. The final Rietveld refinement [15] using the TOPAS program is shown in Fig. S17. Agreement factors are listed in Tab. S1. The atomic coordinates are given in Tab. S2. The crystallographic data have been deposited under number CCDC- 2496867.

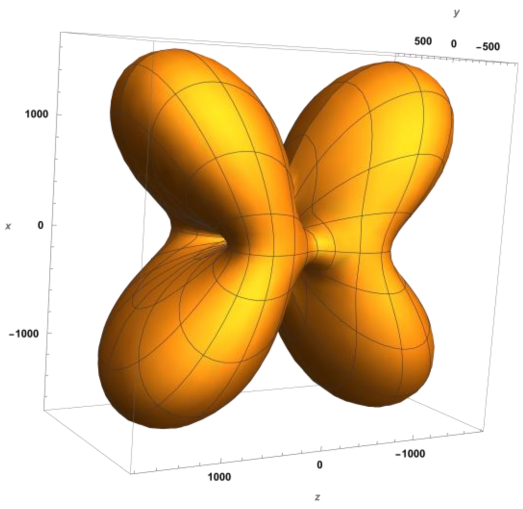

Figure S16. Tensor surface featuring the microstrain distribution in the crystal structure of DBT at ambient conditions.

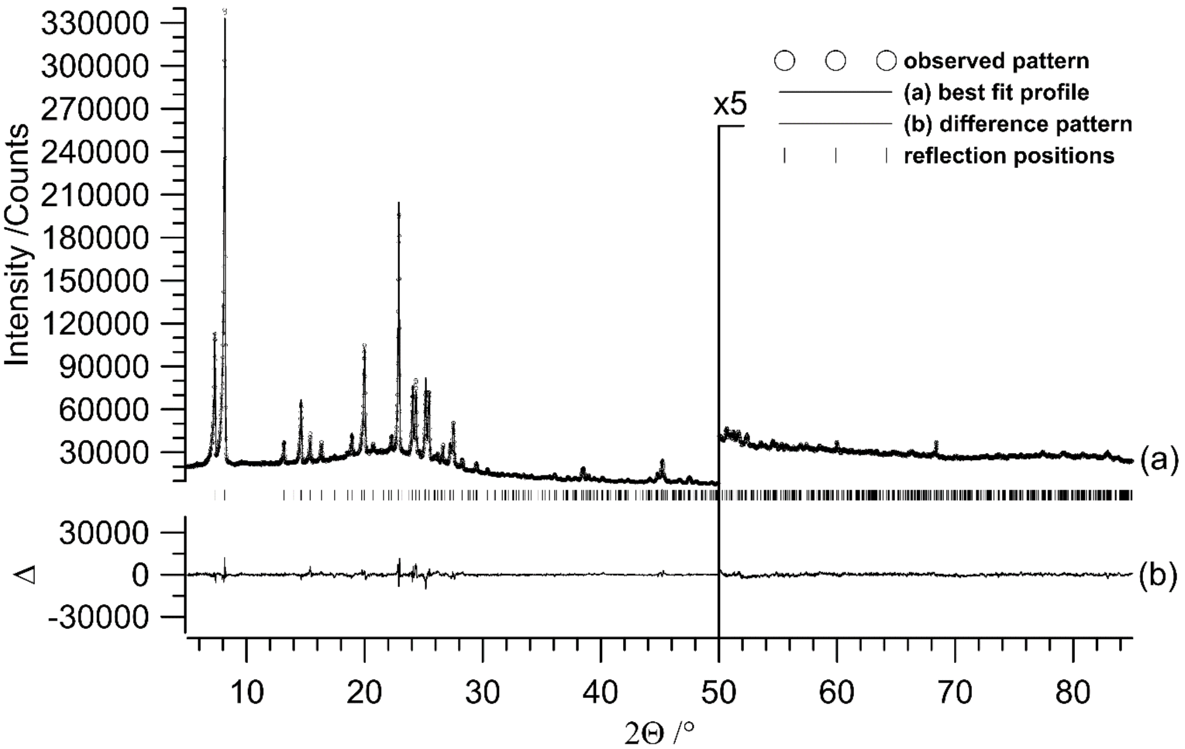

Figure S17. Scattered X-ray (λ = 1.54059 Å) diffraction intensities of DBT at ambient conditions as a function of °2θ. The observed pattern (open circles) measured in Debye-Scherrer geometry, the best Rietveld fit profile (solid line) and the difference curves between the observed and the calculated profiles (solid line in separate window below) are shown. The peak positions of the three phases are presented as vertical lines below the powder pattern. The higher angle part is magnified by a factor of 5 for better visibility.

Table S1. Crystallographic and Rietveld Refinement data of DBT.

**Sum formula**	$C_{38}H_{20}$
**Cell mass/g mol^{-1}**	1906(1)
**Crystal system**	Orthorhombic
**Space group**	*Pbcn*
**Wavelength/ Å**	1.54059
a **/ Å**	24.1912(6)
b**/ Å**	12.1247(3)
c**/ Å**	7.76541(7)
α**/ °**	90
β**/ °**	90
γ**/ °**	90
**V/ Å^3**	2277.69(8)
**T/ K**	298
**Z**	4
**refined parameters**	42
**D_{calc}/g cm^{-3}**	1.3897(1)
**R_{wp}/% [a]**	3.16
**R_p/% [a]**	2.34
**R_{Bragg}/% [a]**	1.70
**Starting angle meas./ ° 2 θ**	2
**Final angle meas./ ° 2 θ**	112
**Starting angle used/ ° 2 θ**	5
**Final angle used/ ° 2 θ**	85
**Step width (° 2 θ)**	0.015
**Time(hrs)**	3

[a]as defined in TOPAS 7.0

Table S2. Atomic coordinates of DBT.

Atom	Multipl.	x/a	y/b	z/c	B	u/Å^2
**C2**	8	-0.07552	0.03525	-0.02173	4.27(8)	0.054
**C3**	8	-0.12645	-0.02346	-0.06319	4.27(8)	0.054
**C4**	8	-0.0732	0.15068	0.00689	4.27(8)	0.054
**C5**	8	-0.17138	0.0239	-0.14731	4.27(8)	0.054
**C7**	8	0.02035	-0.20572	-0.0119	4.27(8)	0.054
**C8**	8	-0.02889	-0.14398	0.01177	4.27(8)	0.054
**C9**	8	-0.02642	-0.02752	-0.00252	4.27(8)	0.054
**C12**	8	-0.08271	-0.19442	0.04325	4.27(8)	0.054
**C13**	8	-0.13056	-0.13602	-0.01247	4.27(8)	0.054
**C14**	8	-0.22157	-0.0329	-0.16508	4.27(8)	0.054
**C15**	8	-0.22809	-0.13635	-0.09577	4.27(8)	0.054
**C16**	8	-0.09011	-0.29626	0.12249	4.27(8)	0.054
**C17**	8	-0.14235	-0.34622	0.13048	4.27(8)	0.054
**C18**	8	-0.18754	-0.29674	0.05599	4.27(8)	0.054
**C19**	8	-0.18279	-0.19046	-0.0175	4.27(8)	0.054
**C25**	8	0.01958	-0.32028	-0.05323	4.27(8)	0.054
**C26**	8	0.06693	-0.37818	-0.08493	4.27(8)	0.054
**C36**	8	-0.11868	0.32426	0.08018	4.27(8)	0.054
**C37**	8	-0.12139	0.21423	0.0438	4.27(8)	0.054
**H39**	8	-0.25568	0.00662	-0.23115	4.27(8)	0.054
**H40**	8	-0.05512	-0.33794	0.17949	4.27(8)	0.054
**H41**	8	-0.14656	-0.42559	0.19306	4.27(8)	0.054
**H42**	8	-0.22744	-0.33744	0.05502	4.27(8)	0.054
**H43**	8	-0.26757	-0.17843	-0.10255	4.27(8)	0.054
**H52**	8	0.06458	-0.46434	-0.12092	4.27(8)	0.054
**H53**	8	-0.01983	-0.36076	-0.06895	4.27(8)	0.054
**H56**	8	-0.15601	0.36906	0.11262	4.27(8)	0.054
**H57**	8	-0.16073	0.17267	0.05227	4.27(8)	0.054
**H58**	8	-0.16774	0.10589	-0.2005	4.27(8)	0.054

Computational details

Table S3. Absolute and relative energies of DBT conformers

Conformer	Total energy, Hartree	Relative energy, kcal/mol
C_{2v}	-1458.9285	3.65
C_{2h}	-1458.9344	0.00
D_2	-1458.9252	5.75
C_1	-1458.9238	6.62

Table S4. Absolute and relative transition state energies between DBT conformers

Transition state	Total energy, Hartree	Transition state energy, kcal/mol	
		Relative to C_{2h} minimum	Relative to C_1 minimum
$C_{2v} \Leftrightarrow C_1$	-1458.916360	11.30	4.68
$C_{2h} \Leftrightarrow C_1$	-1458.917878	10.35	3.72
$D_2 \Leftrightarrow C_1$	-1458.913178	13.30	6.67

Biradical character was calculated based on formulas of Nakano [16]

$$y = 1 - \frac{2T}{1+T^2}; \qquad T = \frac{n_{HOMO} - n_{LUMO}}{2}$$

where *n* is occupation number of UHF natural orbital (UNO)

And UNO occupation obtained from *UHF/6-31+G(d,p) guess=mix Pop=NO* calculation in Gaussian 16C, see table below (just HONO and LUNO were used).

Table S5. UNO occupation

121	122	123	**124**	**125**	126	127	128	129	130
1.77543	1.72122	1.71447	**1.22573**	**0.77427**	0.28553	0.27878	0.22457	0.19299	0.16971

Odd-electron density (OED) was postprocessed by Multiwfn 3.8 [17] from wfn file generated from
LC-UBLYP/6-31+G IOP(3/107=0000003300,3/108=0000003300) guess=mix out=wfn* calculation in Gaussian 16C

Extremely similar OED plot was generated from CASSCF (2x2) calculation performed in ORCA 5.0.3

```
! def2-TZVP MOREAD KeepDens AIM
[…]
%casscf
 Actorbs natorbs
 nel 2 # number of active electrons
 norb 2 # number of active orbitals
 mult 1 # multiplicity blocks
end
```

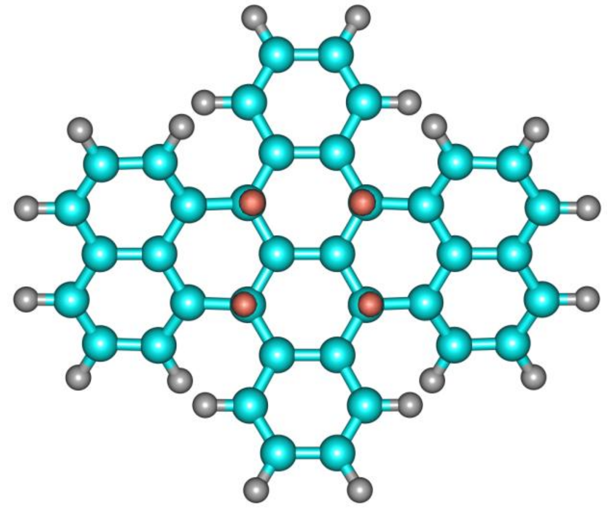	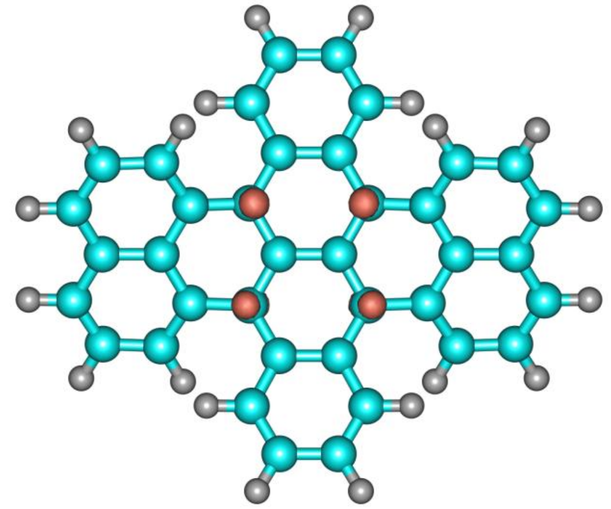
OED plot from LC-UBLYP/6-31+G*<br>Contour value 0.01 e/Bohr^3	OED plot from CASSCF(2x2)/def2-TZVP<br>Contour value 0.002 e/Bohr^3

As it was shown in [18]. FOD plots and triplet state spin densities are often in very good agreement with OED plots (Fig. S18). This was observed also in this case:

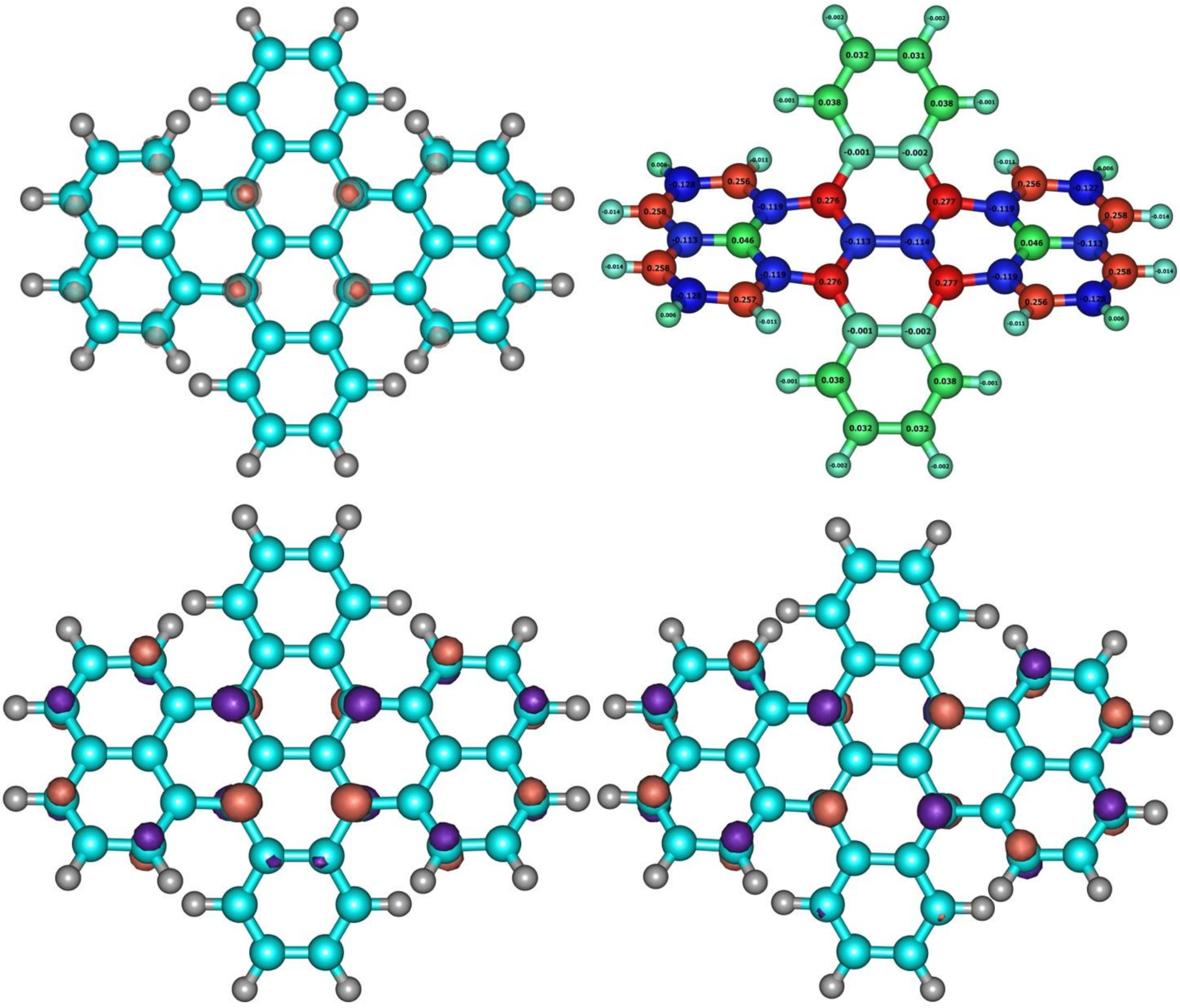

Figure S18. FOD plot of singlet DBT (upper left, solid - contour value 0.009, transparent – contour value 0.005 e/Bohr^3), color map of spin on each atom of DBT in lowest triplet state (upper right), two SOMO orbitals of triplet (contour value 0.06 e/Bohr^3)

XYZ coordinates

C_{2h}

58

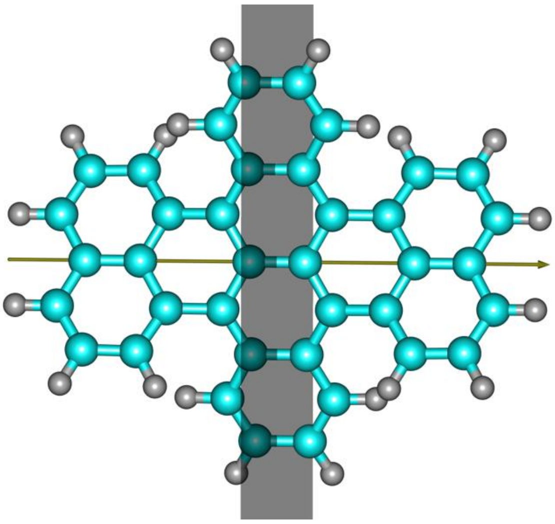

C	2.688041034	-1.134380864	1.071602980
C	0.112463924	1.110584292	-1.515604917
C	-0.778719248	1.536244946	-2.587725432
C	1.480124112	1.477281901	-1.447495042
C	-0.358524995	1.837164235	-3.878952316
C	-1.479263140	-1.480549041	1.443665916
C	-2.355168965	-0.805649078	0.519087956
C	-1.841818878	0.219180983	-0.315022921
C	-0.437458131	0.335658187	-0.464228150
C	1.842696254	-0.222486224	0.311164160
C	2.356025173	0.802399929	-0.522891152
C	-2.687154133	1.131081973	-1.075456978
C	-2.163799950	1.665267885	-2.286326180
C	-1.236972987	2.393262318	-4.818758410
C	-2.534225083	2.686396351	-4.475498591
C	-3.974468280	1.498943242	-0.699060080
C	-4.794416257	2.250619416	-1.551474138
C	-4.355451557	2.616091978	-2.800701283
C	-3.029786347	2.331743346	-3.200169250
C	3.030628268	-2.335189348	3.196248047
C	2.535011969	-2.690002165	4.471512299
C	1.237720185	-2.396993018	4.814731371
C	0.359300769	-1.840817145	3.874943269
C	-0.111586019	-1.113939276	1.511704351
C	-3.698907166	-1.268429928	0.413056181
C	-4.170045312	-2.302969333	1.168219103
C	-3.312093413	-2.964550434	2.072960325
C	-2.011276250	-2.569711180	2.193075094
C	4.356313587	-2.619480300	2.796806009
C	4.795308449	-2.253917248	1.547615907
C	3.975370527	-1.502210912	0.695219840
C	0.779573869	-1.539680365	2.583795232
C	2.164665155	-1.668647211	2.282430377
C	3.699727390	1.265263861	-0.416748251
C	4.170842253	2.299888993	-1.171805338
C	3.312891413	2.961485969	-2.076531213
C	2.012107058	2.566569984	-2.196750230
C	0.438336037	-0.338995179	0.460341872
H	-0.874351102	2.616189347	-5.814136582
H	-4.354063525	1.205122337	0.268070082
H	-5.791626440	2.518064010	-1.225906832
H	-5.008123430	3.143753135	-3.485128024
H	-3.198223995	3.170891990	-5.180628334
H	3.198991302	-3.174558130	5.176620346
H	0.875044853	-2.620085150	5.810052392
H	-0.661424994	-1.642433357	4.165739397
H	5.008969585	-3.147189082	3.481210525
H	5.792529216	-2.521332208	1.222058126
H	4.354980590	-1.208347143	-0.271890212
H	-1.345619955	-3.142117448	2.818629279
H	-3.669395480	-3.821677346	2.629285955
H	-5.185445435	-2.652313103	1.031365929
H	-4.338644359	-0.833746802	-0.337954817
H	4.339439183	0.830593964	0.334290264
H	5.186206391	2.649299967	-1.034856911
H	3.670153261	3.818705486	-2.632742011
H	1.346449176	3.139029169	-2.822241443
H	0.662145204	1.638623281	-4.169819354

C_{2v}

58
symmetry c1

C	2.904183000	0.721092000	0.771580000
C	-0.669969000	0.858630000	-1.539233000
C	-1.975957000	1.330630000	-1.967941000
C	0.551106000	1.155811000	-2.205099000
C	-2.137123000	2.446599000	-2.788898000
C	-0.233099000	-2.096020000	1.430207000
C	-1.480010000	-1.944517000	0.724652000
C	-1.726886000	-0.756206000	-0.016999000
C	-0.591382000	-0.018081000	-0.430266000
C	1.796628000	0.551013000	-0.154477000
C	1.800172000	0.998752000	-1.504462000
C	-3.044706000	-0.303332000	-0.430046000
C	-3.146204000	0.726975000	-1.414419000
C	-3.400981000	2.900218000	-3.176769000
C	-4.534094000	2.280299000	-2.714106000
C	-4.214952000	-0.730060000	0.197861000
C	-5.473787000	-0.268486000	-0.196585000
C	-5.590545000	0.665586000	-1.194642000
C	-4.434483000	1.213949000	-1.793014000
C	3.965463000	0.171271000	2.930824000
C	3.932661000	-0.508353000	4.168749000
C	2.852137000	-1.286132000	4.500308000
C	1.802056000	-1.462474000	3.594860000
C	0.736957000	-1.057692000	1.372693000
C	-2.375112000	-3.050008000	0.705794000
C	-2.094162000	-4.220139000	1.353105000
C	-0.874594000	-4.367815000	2.044960000
C	0.026700000	-3.341140000	2.067430000
C	4.998823000	1.088684000	2.637871000
C	4.942002000	1.850740000	1.498442000
C	3.893868000	1.686876000	0.588321000
C	1.829739000	-0.901396000	2.318040000
C	2.907853000	-0.023453000	1.990909000
C	3.004165000	1.189529000	-2.237793000
C	3.000874000	1.537062000	-3.559221000
C	1.778556000	1.690852000	-4.244769000
C	0.597782000	1.492567000	-3.586575000
C	0.655711000	-0.171851000	0.271167000
H	-3.477189000	3.760283000	-3.829886000
H	-4.153050000	-1.400334000	1.039915000
H	-6.356108000	-0.639779000	0.309036000
H	-6.562187000	1.033447000	-1.500012000
H	-5.516940000	2.630483000	-3.004008000
H	4.746959000	-0.361308000	4.867318000
H	2.794060000	-1.752595000	5.475546000
H	0.931908000	-2.011589000	3.916862000
H	5.803109000	1.216093000	3.351768000
H	5.697522000	2.601685000	1.305359000
H	3.833646000	2.359182000	-0.252233000
H	0.995782000	-3.504568000	2.511667000
H	-0.624579000	-5.315065000	2.505284000
H	-2.781023000	-5.053790000	1.282398000
H	-3.263215000	-2.988105000	0.097119000
H	3.944769000	0.974469000	-1.756096000
H	3.937339000	1.632774000	-4.093647000
H	1.775570000	1.904303000	-5.305935000
H	-0.323858000	1.511950000	-4.146232000
H	-1.270907000	3.006487000	-3.102684000

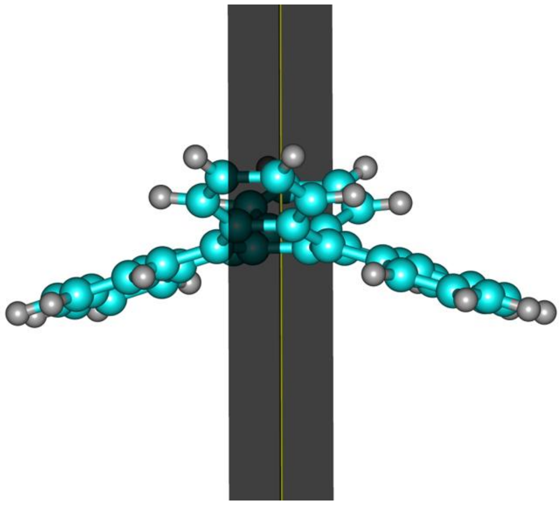

D_2

58
symmetry c1

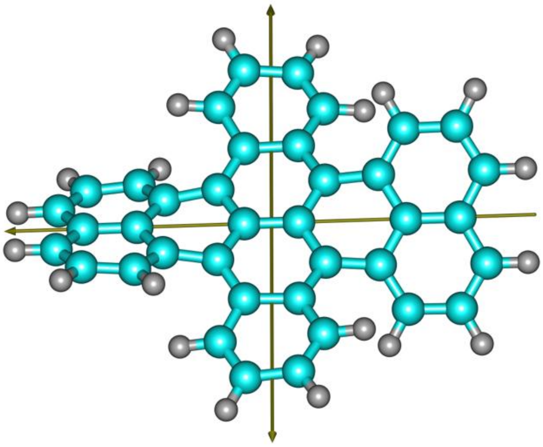

C	2.930551000	0.619614000	0.824096000
C	-0.727026000	0.975548000	-1.439919000
C	-1.971179000	0.964147000	-2.205122000
C	0.387817000	1.772747000	-1.793001000
C	-2.065564000	1.278525000	-3.557681000
C	-0.372442000	-1.829536000	1.739215000
C	-1.661150000	-1.548166000	1.162377000
C	-1.800438000	-0.512030000	0.208245000
C	-0.637181000	0.113757000	-0.314023000
C	1.748568000	0.696258000	-0.030273000
C	1.653303000	1.573535000	-1.136483000
C	-3.097755000	-0.016535000	-0.244098000
C	-3.165377000	0.596101000	-1.525737000
C	-3.308980000	1.401553000	-4.192580000
C	-4.474722000	1.249268000	-3.482761000
C	-4.269605000	-0.088943000	0.503238000
C	-5.508010000	0.268435000	-0.046810000
C	-5.601906000	0.668966000	-1.357120000
C	-4.431952000	0.842042000	-2.130429000
C	4.428334000	-0.827768000	2.141988000
C	4.714597000	-2.117749000	2.643064000
C	3.783469000	-3.121112000	2.532043000
C	2.499346000	-2.852284000	2.038900000
C	0.783663000	-1.183075000	1.240747000
C	-2.745740000	-2.402724000	1.532456000
C	-2.635160000	-3.321770000	2.532446000
C	-1.431752000	-3.411993000	3.270759000
C	-0.337397000	-2.707621000	2.866797000
C	5.351702000	0.238289000	2.228557000
C	5.026494000	1.473129000	1.722828000
C	3.834873000	1.661240000	1.009615000
C	2.139645000	-1.576641000	1.614815000
C	3.164659000	-0.594442000	1.526326000
C	2.793923000	2.225684000	-1.699550000
C	2.681347000	3.155438000	-2.689247000
C	1.399120000	3.534706000	-3.151305000
C	0.300017000	2.846204000	-2.733174000
C	0.639361000	-0.126156000	0.302632000
H	-3.341364000	1.650437000	-5.245639000
H	-4.232539000	-0.418639000	1.529740000
H	-6.398031000	0.195530000	0.565158000
H	-6.564691000	0.879979000	-1.805896000
H	-5.436770000	1.412573000	-3.952444000
H	5.683458000	-2.305830000	3.088882000
H	4.024514000	-4.125521000	2.856054000
H	1.783924000	-3.657958000	1.985505000
H	6.302767000	0.071562000	2.718936000
H	5.706191000	2.307008000	1.843751000
H	3.619783000	2.638289000	0.605974000
H	0.574180000	-2.781713000	3.436777000
H	-1.374477000	-4.048626000	4.144378000
H	-3.470204000	-3.968446000	2.769607000
H	-3.665471000	-2.341664000	0.974094000
H	3.774420000	1.940654000	-1.354855000
H	3.569050000	3.611283000	-3.108625000
H	1.291417000	4.357849000	-3.846016000
H	-0.672613000	3.143636000	-3.089206000
H	-1.169003000	1.426955000	-4.139004000

C_1

58
symmetry c1

C	2.941731000	0.679484000	0.825105000
C	-0.677454000	1.030671000	-1.375454000
C	-2.000225000	1.493004000	-1.756349000
C	0.517507000	1.489255000	-1.980731000
C	-2.260747000	2.769620000	-2.243185000
C	-0.316493000	-2.014569000	1.543401000
C	-1.588412000	-1.784374000	0.911966000
C	-1.702927000	-0.815870000	-0.115804000
C	-0.582648000	0.008429000	-0.399401000
C	1.798402000	0.597899000	-0.070383000
C	1.781169000	1.193205000	-1.361770000
C	-2.892421000	-0.664158000	-0.955922000
C	-3.079637000	0.577166000	-1.631915000
C	-3.531955000	3.117056000	-2.717785000
C	-4.539185000	2.183534000	-2.758700000
C	-3.852230000	-1.652112000	-1.161614000
C	-5.049152000	-1.381611000	-1.838970000
C	-5.320530000	-0.121009000	-2.310270000
C	-4.334016000	0.887298000	-2.232756000
C	4.078703000	-0.047153000	2.888437000
C	4.070410000	-0.788491000	4.089697000
C	2.963251000	-1.519819000	4.433863000
C	1.870395000	-1.600801000	3.566277000
C	0.736313000	-1.074815000	1.394205000
C	-2.696065000	-2.542603000	1.405139000
C	-2.530097000	-3.616915000	2.227475000
C	-1.223990000	-4.035888000	2.574163000
C	-0.162524000	-3.238790000	2.264329000
C	5.127628000	0.857202000	2.612885000
C	5.038672000	1.707022000	1.540346000
C	3.942969000	1.639356000	0.675660000
C	1.876818000	-1.003692000	2.304807000
C	2.976956000	-0.151775000	1.984017000
C	2.964038000	1.447626000	-2.110402000
C	2.926586000	2.034714000	-3.343380000
C	1.688482000	2.395522000	-3.916819000
C	0.524749000	2.117431000	-3.259105000
C	0.654094000	-0.141944000	0.327323000
H	-3.705790000	4.121197000	-3.082982000
H	-3.676510000	-2.655986000	-0.811351000
H	-5.772359000	-2.177186000	-1.966109000
H	-6.269246000	0.104695000	-2.781234000
H	-5.503312000	2.427908000	-3.187222000
H	4.919698000	-0.714152000	4.757468000
H	2.914878000	-2.021868000	5.391787000
H	0.985379000	-2.104131000	3.915797000
H	5.965097000	0.910336000	3.297374000
H	5.802841000	2.454526000	1.369353000
H	3.857721000	2.382346000	-0.100020000
H	0.832513000	-3.555028000	2.534666000
H	-1.069356000	-4.969858000	3.098868000
H	-3.392792000	-4.159151000	2.592800000
H	-3.693291000	-2.233930000	1.139133000
H	3.909104000	1.104687000	-1.719593000
H	3.843131000	2.178317000	-3.901082000
H	1.657654000	2.830812000	-4.907436000
H	-0.416610000	2.299839000	-3.752876000
H	-1.471921000	3.507525000	-2.253721000

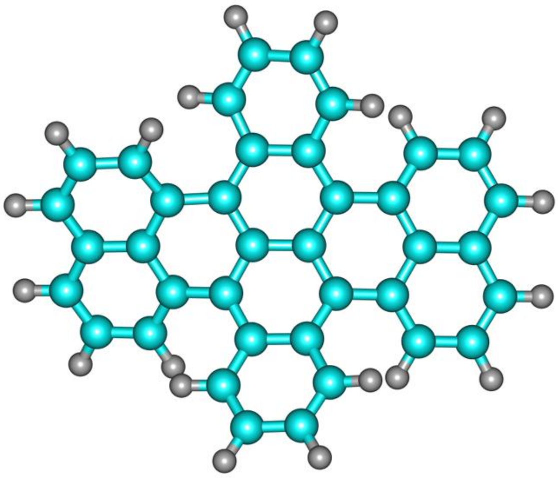

TS C$_{2h}$-C$_1$

58

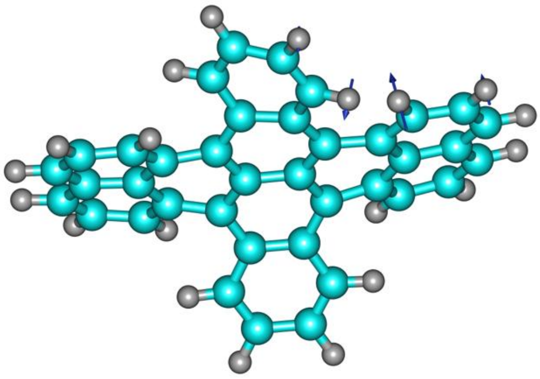

C	2.839764000	0.802023000	0.917102000
C	-0.640674000	0.954034000	-1.442821000
C	-1.956553000	1.381055000	-1.878734000
C	0.552100000	1.450194000	-2.010560000
C	-2.211805000	2.616126000	-2.460447000
C	-0.331999000	-2.011500000	1.586086000
C	-1.605807000	-1.650271000	1.024299000
C	-1.682246000	-0.788681000	-0.088484000
C	-0.546756000	-0.027043000	-0.426461000
C	1.766358000	0.638020000	-0.032156000
C	1.777601000	1.277647000	-1.294359000
C	-2.870940000	-0.687625000	-0.924823000
C	-3.040965000	0.485571000	-1.703968000
C	-3.472207000	2.926180000	-2.980100000
C	-4.479516000	1.996110000	-2.966408000
C	-3.842771000	-1.675461000	-1.024677000
C	-5.031931000	-1.457863000	-1.728180000
C	-5.279902000	-0.248765000	-2.323824000
C	-4.285330000	0.750536000	-2.334697000
C	4.191524000	-0.115960000	2.721887000
C	0.821593000	-1.217996000	1.303277000
C	-2.791098000	-2.151343000	1.637472000
C	-2.764557000	-3.134081000	2.574809000
C	-1.525380000	-3.731962000	2.872802000
C	5.014860000	1.027519000	2.661651000
C	4.713189000	2.059863000	1.815578000
C	3.631150000	1.943639000	0.943630000
C	2.113109000	-1.295196000	2.022919000
C	3.043033000	-0.213609000	1.882453000
C	2.984020000	1.692311000	-1.920585000
C	2.990361000	2.260142000	-3.159569000
C	1.780685000	2.439031000	-3.860159000
C	0.603055000	2.039527000	-3.302486000
C	0.684489000	-0.207144000	0.303748000
H	-3.638288000	3.902015000	-3.421844000
H	-3.680739000	-2.638297000	-0.560263000
H	-5.768948000	-2.251141000	-1.778498000
H	-6.224217000	-0.057780000	-2.821936000
H	-5.438948000	2.209653000	-3.424749000
H	5.872978000	1.082116000	3.322652000
H	5.312560000	2.962881000	1.811193000
H	3.391365000	2.767533000	0.285094000
H	-1.479886000	-4.622261000	3.489671000
H	-3.676550000	-3.470386000	3.053193000
H	-3.732976000	-1.697397000	1.362703000
H	3.923244000	1.487163000	-1.425388000
H	3.931274000	2.525150000	-3.627763000
H	1.792711000	2.842327000	-4.866191000
H	-0.307573000	2.101170000	-3.882287000
H	-1.425400000	3.357899000	-2.506619000
H	1.867926000	-3.135544000	3.117993000
H	3.864779000	-3.044541000	4.378142000
H	0.530108000	-3.721815000	2.627952000
C	2.483606000	-2.281612000	2.933739000
C	3.652210000	-2.227813000	3.697794000
C	-0.369715000	-3.184769000	2.403936000
C	4.490842000	-1.154677000	3.620940000
H	5.377530000	-1.082271000	4.240801000

TS C_{2v}-C_1

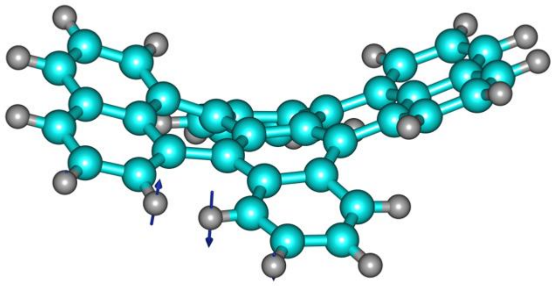

58
symmetry c1

C	2.934749000	0.688701000	0.744347000
C	-0.592805000	0.805676000	-1.556374000
C	-1.869944000	1.353423000	-1.928564000
C	0.617634000	1.151916000	-2.200350000
C	-1.987678000	2.608949000	-2.513315000
C	-0.199449000	-2.075886000	1.462819000
C	-1.446221000	-2.013135000	0.740439000
C	-1.679705000	-0.941730000	-0.183525000
C	-0.546174000	-0.140833000	-0.509319000
C	1.839450000	0.495620000	-0.187201000
C	1.864371000	0.936448000	-1.531740000
C	-3.228215000	3.119210000	-2.892543000
C	3.928102000	0.261458000	2.956997000
C	3.838506000	-0.324323000	4.235724000
C	2.730044000	-1.048454000	4.584655000
C	1.714032000	-1.274111000	3.654897000
C	0.753171000	-1.036417000	1.368209000
C	4.987055000	1.134200000	2.636883000
C	4.975709000	1.818127000	1.450767000
C	3.946155000	1.620776000	0.529615000
C	1.806962000	-0.818602000	2.343471000
C	2.901443000	0.021266000	2.001047000
C	3.060563000	1.118010000	-2.273704000
C	0.686440000	-0.214399000	0.230338000
H	4.632663000	-0.142381000	4.951575000
H	2.623721000	-1.435523000	5.591503000
H	0.818604000	-1.784363000	3.980278000
H	5.774063000	1.294780000	3.365530000
H	5.751944000	2.541715000	1.229415000
H	3.916945000	2.248203000	-0.349103000
H	4.002199000	0.838824000	-1.821164000
H	-4.175485000	-2.319315000	-0.363983000
H	-6.224670000	-1.526172000	-1.220795000
H	-6.396985000	0.713931000	-2.276370000
H	-3.326282000	-3.103485000	0.598345000
H	-2.761126000	-4.942345000	1.939451000
H	-0.450834000	-5.175799000	2.889580000
H	-5.325464000	2.726876000	-3.033908000
H	-3.290870000	4.104276000	-3.340242000
H	-1.100379000	3.211482000	-2.654446000
H	-0.293340000	1.718075000	-4.073256000
H	1.803988000	2.113440000	-5.250029000
H	3.972356000	1.637704000	-4.119297000
C	-4.134722000	-1.346616000	-0.806123000
C	-5.350950000	-0.892616000	-1.322606000
C	-2.951030000	-0.612565000	-0.863602000
C	-5.449108000	0.333555000	-1.912497000
C	-4.286210000	1.106849000	-2.076391000
C	-4.355860000	2.365450000	-2.709309000
C	-3.033574000	0.610767000	-1.610518000
C	-2.344181000	-3.084711000	1.018335000
C	-2.025758000	-4.158534000	1.797464000
C	-0.745640000	-4.278370000	2.358943000
C	0.126755000	-3.247862000	2.198663000
H	1.131397000	-3.337475000	2.587048000
C	1.817220000	1.812143000	-4.209035000
C	0.644060000	1.608917000	-3.544719000
C	3.043483000	1.548152000	-3.567846000

TS D_2-C_1

58

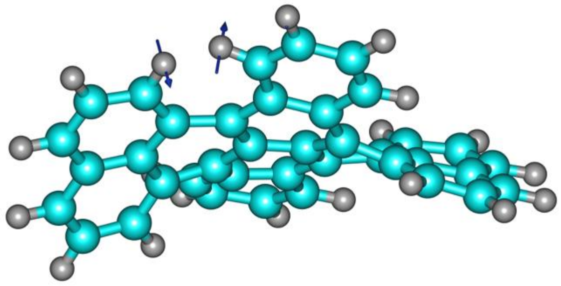

C	2.912196000	0.551070000	0.832445000
C	-0.780610000	1.058035000	-1.306895000
C	-1.905595000	0.938044000	-2.206270000
C	0.275602000	1.963893000	-1.542374000
C	-1.810619000	1.259727000	-3.555061000
C	-0.401006000	-1.710859000	1.839257000
C	-1.727845000	-1.285974000	1.461380000
C	-1.898635000	-0.447762000	0.312801000
C	-0.724654000	0.179498000	-0.199990000
C	1.681564000	0.782707000	0.082034000
C	1.542409000	1.789251000	-0.896584000
C	-3.170880000	-0.183034000	-0.393717000
C	-3.109813000	0.386305000	-1.707469000
C	-2.917226000	1.190302000	-4.400850000
C	-4.133990000	0.819227000	-3.896482000
C	-4.257313000	0.409613000	-2.552916000
C	4.444224000	-1.142708000	1.754820000
C	4.694418000	-2.501376000	2.039490000
C	3.692757000	-3.429510000	1.916572000
C	2.386070000	-3.028389000	1.617630000
C	0.737074000	-1.179735000	1.199495000
C	-2.574086000	-2.604546000	3.355315000
C	-1.278531000	-3.010593000	3.708691000
C	-0.231288000	-2.559777000	2.968263000
C	5.428279000	-0.144165000	1.907540000
C	5.117190000	1.165335000	1.650867000
C	3.876051000	1.512325000	1.103362000
C	2.084238000	-1.695408000	1.381106000
C	3.149290000	-0.763971000	1.313794000
C	2.623984000	2.589995000	-1.371143000
C	2.434785000	3.615261000	-2.245301000
C	1.132222000	3.937769000	-2.685199000
C	0.098636000	3.113074000	-2.367807000
C	0.571321000	-0.050905000	0.375942000
H	-2.808747000	1.445703000	-5.448532000
H	-5.017785000	0.806131000	-4.524925000
H	5.692142000	-2.799547000	2.342769000
H	3.899627000	-4.479755000	2.087067000
H	1.601948000	-3.772944000	1.568868000
H	6.413790000	-0.423084000	2.263999000
H	5.846507000	1.944893000	1.839390000
H	3.671671000	2.554149000	0.901391000
H	0.774862000	-2.786915000	3.287744000
H	-1.109235000	-3.624537000	4.585356000
H	-3.424844000	-2.915437000	3.950952000
H	3.628714000	2.349351000	-1.060296000
H	3.284774000	4.190278000	-2.594551000
H	0.960034000	4.823140000	-3.286071000
H	-0.898747000	3.349446000	-2.712382000
H	-0.853174000	1.551070000	-3.964360000
H	-4.635477000	-0.849624000	1.046931000
H	-3.783814000	-1.447059000	2.152266000
C	-4.452060000	-0.499250000	0.053182000
C	-2.775519000	-1.772231000	2.293712000
C	-5.595669000	-0.385682000	-0.742297000
H	-6.553522000	-0.657515000	-0.313675000
C	-5.507682000	0.017105000	-2.042736000
H	-6.379992000	0.052771000	-2.685760000

References

1. S. Stoll, A. Schweiger, *J. Magn. Reson.* ***2006***, 178, 42-55

2. Clar, E.; Willicks, W. Aromatische Kohlenwasserstoffe, LXIX. Mitteil.: 7.8;15.16-Dibenzterrylen. Chem. Ber. **1955**, 88, 1205–1207, doi:10.1002/cber.19550880809.

3. F. Tampieri, S. Silvestrini, R.Ricc`o, M. Maggini and A. Barbon, *J. Mater. Chem. C,* **2014**, 2, 8105.

4. D. Keeble and B. Ramakrishnan, *Appl. Phys. Lett*., **1996**, 69, 3836–3838.

5. A. M. Panich, A. I. Shames, M. I. Tsindlekht, V. Yu. Osipov, M. Patel, K. Savaram and H. He, *J. Phys. Chem. C* **2016**, 120, 5, 3042–3053

6. C. Erker, T. Basché. The Energy Gap Law at Work: Emission Yield and Rate Fluctuations of Single NIR Emitters. Journal of the American Chemical Society, **2022**, 144(31), 14053–14056. https://doi.org/10.1021/jacs.2c07188

7. Coelho, A. A. J. Appl. Crystallogr. **2018**, 51, 210-218.

8. Coelho, A.A. J. Appl. Cryst, **2003**, 36, 86-95.

9. Pawley, G. S. J. Appl. Cryst. **1981**, 14, 357-361.

10. Cheary, R. W., Coelho, A. A., Cline,J. P. J. Res. Natl. Inst. Stand. Technol. **2005**, 109, 1-25.

11. Black, D. R., Mendenhall, M. H., Brown, C. M., Henins, A., Filliben, J., Cline, J. P. Powder Diffr. **2020**, 35(1).

12. Thompson, P., Cox. D. E., Hastings, J. B. J. Appl. Cryst. **1987**, 20, 79-83.

13. Stephens, P. W. J. Appl. Cryst. **1999**, 32, 281-289.

14. Coelho, A. A. Acta Cryst. **2007**, A36, 400.

15. Rietveld, H. M. J. Appl. Crystallogr. **1969**, 2, 65-71.

16. Kamada, K.; Ohta, K.; Shimizu, A.; Kubo, T.; Kishi, R.; Takahashi, H.; Botek, E.; Champagne, B.; Nakano, M. Singlet Diradical Character from Experiment. J. Phys. Chem. Lett. **2010**, 1, 937–940, doi:10.1021/jz100155s.

17. Lu, T.; Chen, F. Multiwfn: A Multifunctional Wavefunction Analyzer. J. Comput. Chem. **2012**, 33, 580–592, doi:10.1002/jcc.22885.

18. Feofanov, M.; Akhmetov, V.; Sharapa, D.I.; Amsharov, K. Modular Approach to the Synthesis of Two-Dimensional Angular Fused Acenes. Org. Lett. **2020**, 22, 1698–1702, doi:10.1021/acs.orglett.9b04382.
